# Geostatistical analysis of active human cysticercosis: Results of a large-scale study in 60 villages in Burkina Faso

**DOI:** 10.1371/journal.pntd.0011437

**Published:** 2023-07-26

**Authors:** Veronique Dermauw, Ellen Van De Vijver, Pierre Dorny, Emanuele Giorgi, Rasmané Ganaba, Athanase Millogo, Zékiba Tarnagda, Assana Kone Cissé, Hélène Carabin

**Affiliations:** 1 Department of Biomedical Sciences, Institute of Tropical Medicine, Antwerp, Belgium; 2 Department of Environment, Faculty of Bioscience Engineering, Ghent University, Ghent, Belgium; 3 Centre for Health Informatics, Computing, and Statistics, Lancaster University, Lancaster, United Kingdom; 4 AFRICSanté, Bobo-Dioulasso, Burkina Faso; 5 Centre Hospitalier Universitaire Sourô Sanou, Bobo-Dioulasso, Burkina Faso; 6 Université Ouagadougou, Joseph Ki-Zerbo, Ouagadougou, Burkina Faso; 7 Institut de Recherche en Sciences de la Santé, Bobo-Dioulasso, Burkina Faso; 8 Department of Pathology and Microbiology, University of Montreal, Montreal, Canada; 9 Centre de Recherche en Santé Publique (CReSP), Montreal, Canada; 10 Groupe de Recherche en Épidémiologie des Zoonoses et Santé Publique (GREZOSP), Montreal, Canada; 11 Department of Social and Preventive Medicine, University of Montreal, Montreal, Canada; 12 Department of Biostatistics and Epidemiology, University of Oklahoma Health Sciences Center, Oklahoma City, Oklahoma, United States of America; Pontificia Universidad Catolica de Chile, CHILE

## Abstract

Cysticercosis is a neglected tropical disease caused by the larval stage of the zoonotic tapeworm (*Taenia solium*). While there is a clear spatial component in the occurrence of the parasite, no geostatistical analysis of active human cysticercosis has been conducted yet, nor has such an analysis been conducted for Sub-Saharan Africa, albeit relevant for guiding prevention and control strategies. The goal of this study was to conduct a geostatistical analysis of active human cysticercosis, using data from the baseline cross-sectional component of a large-scale study in 60 villages in Burkina Faso. The outcome was the prevalence of active human cysticercosis (hCC), determined using the B158/B60 Ag-ELISA, while various environmental variables linked with the transmission and spread of the disease were explored as potential explanatory variables for the spatial distribution of *T*. *solium*. A generalized linear geostatistical model (GLGM) was run, and prediction maps were generated. Analyses were conducted using data generated at two levels: individual participant data and grouped village data. The best model was selected using a backward variable selection procedure and models were compared using likelihood ratio testing. The best individual-level GLGM included precipitation (increasing values were associated with an increased odds of positive test result), distance to the nearest river (decreased odds) and night land temperature (decreased odds) as predictors for active hCC, whereas the village-level GLGM only retained precipitation and distance to the nearest river. The range of spatial correlation was estimated at 45.0 [95%CI: 34.3; 57.8] meters and 28.2 [95%CI: 14.0; 56.2] km for the individual- and village-level datasets, respectively. Individual- and village-level GLGM unravelled large areas with active hCC predicted prevalence estimates of at least 4% in the south-east, the extreme south, and north-west of the study area, while patches of prevalence estimates below 2% were seen in the north and west. More research designed to analyse the spatial characteristics of hCC is needed with sampling strategies ensuring appropriate characterisation of spatial variability, and incorporating the uncertainty linked to the measurement of outcome and environmental variables in the geostatistical analysis.

Trial registration: ClinicalTrials.gov; NCT0309339.

## Author summary

Cysticercosis is a serious, yet neglected disease caused by the larval stage of a zoonotic tapeworm, prevalent in many developing countries, including Burkina Faso. Being able to predict where the disease occurs is essential for running targeted prevention and control activities. In our study, we aimed to describe whether human cysticercosis cases in three provinces in Burkina Faso were clustered, and investigated whether there was a link between this clustering and some land and weather variables. Finally, we aimed to generate high-resolution prediction maps for the occurrence of the infection. We found that there was clustering at 45 meters for the individual- and 28.2 km for the village-level datasets, respectively. Increasing rainfall and proximity to a river were linked with this clustering. The generated prediction maps indicated there were important cysticercosis hotspots in the study area, especially in the extreme south and north-west, where the disease is thought to be more important. Further research should expand the use of spatial techniques to predict the occurrence of cysticercosis, the results of which can aid in the design of intervention programmes.

## Introduction

Cysticercosis is a neglected tropical zoonosis, acquired by ingestion of eggs shed by a human tapeworm (*Taenia solium*) carrier. Humans can become infected because of poor hand hygiene (faecal-oral route) or through the consumption of contaminated food or water [1,2]. Upon ingestion of the eggs, the larval forms of *T*. *solium* will migrate throughout the body and develop into cysticerci, fluid-filled cyst-like structures (human cysticercosis (hCC)). In humans, cysticerci have a distinct tropism for subcutaneous tissues and the central nervous system (neurocysticercosis (NCC)) [3]. Neurocysticercosis has an important public health impact, as many cases suffer from its neurological manifestations, such as seizures, chronic headaches, increased intracranial pressure and even death [4].

A geostatistical analysis uses a specific set of methods to process georeferenced data that are inherently more similar when closer together. As such, these methods allow researchers to describe and model the spatial variability patterns of the data. Another important, if not the most important, objective of a geostatistical analysis is to predict values at unsampled locations through the creation of continuous surfaces from point data, also called interpolation, while also estimating the uncertainty linked to these predictions [5]. These techniques can be used for public health threats, to unravel the spatial structure of the variability in prevalence estimates, to identify environmental characteristics affecting the distribution of prevalence estimates, and to predict where a prevalence value exceeds a specific threshold [6]. The latter can be most useful in terms of the identifying target areas for intervention strategies.

Model-based geostatistical methods have been used for a number of neglected tropical diseases such as lymphatic filariasis [7], onchocerciasis [8,9], schistosomiasis [10–14] and soil-transmitted helminth infections [15–18]. Studies focussing on the spatial distribution of the infections caused by *T*. *solium* have so far unravelled relevant information about disease clustering [19–24] and distance-dependent relationships between conditions caused by the different life stages of the parasite [25–28]. Yet, up to now, only one study has conducted a geostatistical analysis for human infections caused by *T*. *solium*. Using data from a national baseline serosurvey in Colombia, Galipó et al. [29] determined that there was spatial correlation in seropositivity estimated up to approximately 140 km. However, that study used an ELISA applied to blood samples collected on filter paper to detect circulating *T*. *solium* cysticercus antibodies, its results thus point to exposure to the parasite. To our knowledge, no study has so far conducted a geostatistical analysis for active *T*. *solium* infections, nor has such an analysis been conducted for Sub-Saharan Africa. Thus, the aim of this work was to conduct a geostatistical analysis of active hCC, using data from 60 villages in three provinces of Burkina Faso.

## Methods

### Ethics statement

Ethical approval was obtained from the University of Oklahoma Health Sciences Center Institutional Review Board and the Centre MURAZ ethical review panel in Burkina Faso. The data used for this study were collected for a trial, registered with ClinicalTrials.gov, number NCT0309339. Written informed consent was obtained for all study participants. Parents consented in writing for children younger than 18 years and children older than 10 years were asked for their written assent.

### Study design

The field data used in this study were collected in the context of a large cluster randomized-control trial (cRCT), called EFECAB, investigating the effect of an educational intervention on cumulative incidence of active hCC [30–33]. More specifically, currently reported data were collected during the baseline cross-sectional component of this trial, conducted between February 2011 and January 2012 (STROBE checklist, S1 STROBE checklist).

**Fig 1:**
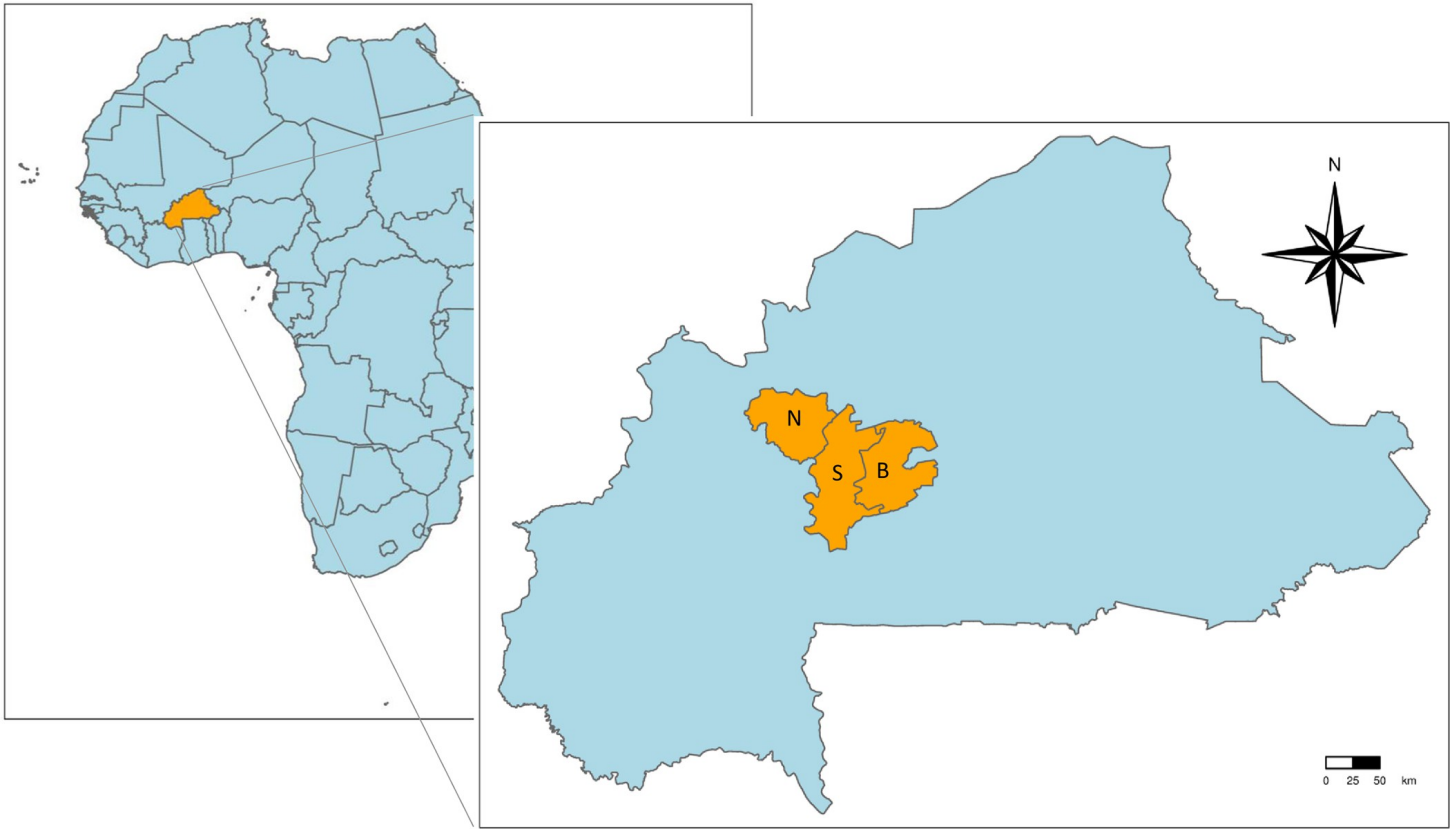
Location of Burkina Faso in Africa, and of the study area in Burkina Faso. (Study provinces: N = Nayala, S = Sanguié, B = Boulkiemde) (https://www.diva-gis.org/gdata).

The study was conducted in 60 villages in three provinces of Burkina Faso (Fig 1). The inclusion and exclusion criteria, selection procedures for study provinces, departments, villages, concessions (i.e., a group of households living in a compound), households, and participants, and rationale for sample sizes, have been described elsewhere [30–33]. Briefly, the study provinces, corresponding together to 4.9% of the country’s total area, were selected due to their large pig population (Boulkiemdé and Sanguié) or neighbouring position and local reports of humans feeding stool to pigs (Nayala). All pig raising departments were included in the study, and in each department, two villages meeting the eligibility criteria for the cRCT namely having a population of at least 1000 people (2006 census), being present on the map of the *Institut Géographique du Burkina* (year 2000), and separated from the other selected villages by at least 5 km, were randomly selected. Capitals of the region or province were excluded as well as villages located within 20 km of the large cities Koudougou or Ouagadougou or on a national or provincial road.

### Participants

In each village, concessions were selected using a stratified random sampling approach, with the different pig production types (reproductive sows, piglets or no pigs) as strata [30]. In each village, 80 concessions were randomly selected (at least 10 concessions raising sows, at least 30 concessions raising piglets aged 12 months or younger). A handheld GPS device was used to record the geographical coordinates at the centre of each concession. After asking the chief of each selected concession for consent to participate in the study, one household per concession was randomly sampled and the household chief asked for consent to participate. Finally, one eligible person from the selected household was randomly selected and asked for consent to participate. Individuals were eligible if they were at least 5 years old, had resided in the village during the past year, and intended to remain there for the next three years.

Eligible individuals (one person per household) were invited to participate in the study, this entailed providing a blood sample for the serological component of the study and answering a screening questionnaire. Recruitment continued until 60 people consented to participate in each village. Additionally, 20 eligible individuals per village, who refused to participate to the serological compound, were invited to only participate in the screening questionnaire. The screening questionnaire investigated the presence of symptoms that could be linked to NCC (i.e. chronic worsening severe headaches, seizures and/or epilepsy) as well as socio-demographic information and knowledge about cysticercosis and epilepsy. Participants who screened positive to the presence of symptoms suggestive of NCC were invited to provide a blood sample to help with the diagnosis of NCC. Parents consented for children younger than 18 years and children older than 10 years were asked for their assent.

### Outcome data

Each of the participants was sampled for blood by means of venipuncture of the antebrachium vein, using a syringe and 10 ml serum gel tubes. Once collected, the tubes were cooled and transported to the processing unit. There, serum was collected, and stored at -20°C until analysis.

Using the B158/B60 Ag-ELISA, serum samples were tested for the presence of excretory/secretory circulating antigens of *T*. *solium* cysticerci, i.e. active cysticercosis [34]. The optical density (OD) of each serum sample was compared with the mean OD of eight negative reference human sera samples at a probability level of *p* = 0.001 to determine the test result. In an earlier study conducted in Ecuador, this B158/B60 Ag-ELISA was reported to have a sensitivity of 90% (95% Bayesian Credible Interval (BCI): 80–99%) and a specificity of 98% (95%BCI: 90–99%) to detect active infection [35]. The Ag-ELISA result (i.e. positive or negative), represented the occurrence of active hCC, and the prevalence of active hCC was considered the outcome variable of the study. All hCC survey data were entered in an Excel file.

### Environmental data

A list of environmental variables considered potentially linked with the transmission and spread of hCC based on earlier work on *Taenia* spp. and other helminths [36] was drafted and explored as potential explanatory variables for the spatial distribution of *T*. *solium*. Datasets for the environmental variables were retrieved from online accessible data sources (Tables 1 and S1). In case datasets for multiple time points were available, it was opted to go for a file that contained data from around the mid-point of the data collection period (i.e. July 2011).

**Table 1:** Sources for the environmental data considered as potential explanatory variables for the spatial distribution of *T*. *solium*.

Variable	Source	Date or time period	Unit	Spatial resolution
Country/province boundary	DIVA-GIS	1992	-	-
Potential evapotranspiration	CGIAR-CSI	July 2011	mm/month	1km
Elevation	CGIAR SRTM	-	m	1km
Land cover	MODIS-Terra	2011	-	0.5km
Land surface temperatures	MODIS-Terra	04-July-2011	Celsius	1km
NDVI	MODIS-Terra	12-July-2011	NDVI	1km
Precipitation	WorldClim	1970–2000, July	mm/month	1km
Water lines	DIVA-GIS	1992	-	-
Soil pH (0–5 cm)	ISRIC	-	-	250m
Soil sand (0–5 cm)	ISRIC	-	g/100g (%)	250m
Soil clay (0–5 cm)	ISRIC	-	g/100g (%)	250m
Soil silt (0–5 cm)	ISRIC	-	g/100g (%)	250m

CGIAR-CSI: Consultative Group for International Agricultural Research—Consortium for Spatial Information (CGIAR-CSI); CGIAR SRTM: CGIAR Shuttle Radar Topography Mission; DIVA-GIS: Data-Interpolating Variational Analysis—Geographic Information System; ISRIC: International Soil Reference and Information Centre; MODIS: Moderate Resolution Imaging Spectroradiometer; NDVI: Normalized Difference Vegetation Index; WorldClim: World Climate

The environmental datasets were clipped to the boundaries of the three study provinces to ensure faster processing of files. The coordinate reference system of the country boundaries and all environmental datasets were converted to the Universal Transverse Mercator 30N zone to allow distance measurements to be expressed in meters. The file with existing waterlines was additionally used to create a dataset (1 km × 1 km) containing distance at each point to the nearest river. Furthermore, a prediction grid with cells of 1 km × 1 km was generated.

Finally, environmental data were extracted at the living locations of the participants to the hCC serosurvey and at the centroids of the prediction grid. For continuous variables, this entailed bilinear interpolation, based on the values of the four nearest cells, while for categorical variables, simple interpolation was chosen, i.e. the value for the cell where a point was included. Values were expressed in their original units by multiplying with the scale factor mentioned at each data source, temperatures were additionally converted from Kelvin to Celsius.

### Data analysis

For the data analysis, two databases were used: i) a dataset including information at the individual level, ii) a dataset including the individual data grouped at the village level. The village-level dataset included the number of sampled individuals and the number of the participants testing positive using the Ag-ELISA per village, the mean coordinates of the village survey points, and the village mean of survey point values for the environmental variables.

Descriptive statistics were calculated for the outcome and environmental variables. Maps of the study area, location of the survey points and village prevalence estimates were created, so were maps of the study area for the environmental data. Spearman’s *ρ*_*s*_ correlation coefficients were calculated to measure the association between sets of two environmental variables to detect potential issues of multicollinearity.

A variogram $\hat{V}(u)$ was calculated to describe the spatial continuity of the outcome variable both at the village- and individual-level as follows:

For the individual-level data, the variogram was calculated using the outcome value for each participant. For the village-level data, the empirical logit [37] of the outcome variable was first calculated:

$$y_{i}^{*}=\log\left(\frac{y_{i}+0.5}{n_{i}-y_{i}+0.5}\right)$$ (1)

with *y*_*i*_, the number of participants with positive test results in village *i*, and *n*_*i*_, the total number of participants samples in village *i*. The addition of 0.5 was included to accommodate for *y*_*i*_ = 0 and *y*_*i*_ = *n*_*i*_, i.e. to avoid plus and minus infinity. The variogram $\hat{V}(u)$ was then calculated for the empirical logit $y_{i}^{*}$, using the following formula (6):

$$\hat{V}(u)=\frac{1}{2|N(u)|}\sum_{\left(h,k)\in N(u\right)}{\left(y_{h}^{*}-y_{k}^{*}\right)}^{2}$$ (2)

with *N*(*u*) being the number of data-pairs at lag distance *u* apart from each other; *u* is the lag distance between two points, and $y_{h}^{*}$ and $y_{k}^{*}$ are the empirical logit for the outcome variable at location *h* and *k*, respectively. Data pairs were first classified in distance bins and the empirical variogram was averaged for each bin.

For both datasets, a variogram model was then fitted to get a first estimate for the variogram parameters, using weighted least squares, with the weights being the number of pairs per bin, and the general-purpose Nelder–Mead approach for numerical optimization [38].

Next, and for each dataset, the association between the outcome variable and each environmental variable was explored, using scatter plots and generalized linear models (GLM) (for detailed procedure see S1 Text). Briefly, the final selection of environmental variables was done using a backward stepwise approach, where models were compared using the likelihood-ratio test. Once the final model had been selected, the presence of residual spatial correlation was investigated using a using a Monte Carlo method (see S1 Text for details).

The selected environmental variables were then included in a generalized linear geostatistical model (GLGM) formulated with the following structure (6):

$$log\left\{\frac{p(x_{i})}{1-p(x_{i})}\right\}=d{\left(x_{i}\right)}^{t}\beta +S(x_{i})+Z_{i}$$ (3)

with *p*(*x*_*i*_) being the probability for a positive test result; *d*(*x*_*i*_)^*t*^*β*, the transposed column vector of the selected explanatory variables *d*_*i*_ at point *x*_*i*_ with regression coefficients *β*. The spatial term, *S*(*x*_*i*_)~*N*(0,*σ*^2^) represents a dependent stationary isotropic Gaussian process, i.e. a Gaussian process with constant variance (*σ*^2^ compares to the sill in classical geostatistical terminology) of which the covariance only depends on the distance between points. The correlation between *S*(*x*_*i*_) and *S*(*x*_*j*_), the spatial component at locations *i* and *j*, is defined by the correlation function *ρ*(*u*). Several correlation functions have been suggested including the exponential, spherical and Matérn families. Here, we assume that $\rho (u;\phi )=exp(-\frac{u}{\phi })$, where *u* is the lag distance between points *x*_*i*_ and *x*_*j*_, with *u*≥0; and *ϕ* a scale factor with *ϕ*>0. For this exponential correlation function, the practical range *u*_*p*_ (i.e. the lag distance where 95% of the sill is reached, or where *ρ*(*u*) has decayed to 0.05) is *u*_*p*_≈3×*ϕ*. *Z*_*i*_ is a mutually independent Gaussian process, i.e. the spatial variation at a distance below the minimum observed distance, or random variation due to measurement error, with *Z*_*i*_~*N*(0, *τ*^2^), where *τ*^2^ compares to the nugget in classical geostatistical terminology.

Three GLGMs were fitted, ℳ_1_ and ℳ_2_, an individual- and a village- level model, respectively, with the covariates selected in the final individual and village level GLM (S3 Table) as well as an additional GLGM, ℳ_3_, which had the same covariates as the village-level GLM, to allow comparison of the final prediction maps generated by the village- and individual-level data.

Parameter estimation was performed using the Monte Carlo maximum-likelihood (MCML) method which is detailed in [39] and [40]. The algorithm used to fit the GLGM (ℳ_1_, ℳ_2_, ℳ_3_) had the GLM estimates for the coefficients (S3 Table), and the weighted least squares estimates for the variogram parameters *σ*^2^ and *ϕ*, as starting values, while *τ*^2^ was fixed at 0 (as estimated as such in the variogram analysis). Overfitting was assessed by investigating the correlation matrix of the regression coefficient estimates, with a Pearson’s *ρ* correlation coefficient approaching 1 or -1 considered indicative for overfitting [41].

The predictive target, T*, was defined as the prevalence surface over the study area, *A* (6):

$$T^{*}=\left\{p(x)=\frac{\exp\left\{T(x)\right\}}{1+\exp\left\{T(x)\right\}}:x\in A\right\}$$ (4)

with *p*(*x*) being the probability for a positive test result; and *T*(*x*) = *d*(*x*)^*t*^*β*+*S*(*x*). Maps of the predicted prevalence and associated standard errors were created.

Additionally, both for the individual-level and village-level datasets, maps were generated for the exceedance of a set prevalence threshold, *l*. Based on our own experience, we set the prevalence threshold was at 5%, as this prevalence was deemed worth identifying for prioritising intervention campaigns. The probability for the prevalence to exceed this threshold (also called the 5% exceedance probability) was calculated as follows (6):

$$R_{l}(T^{*})=\{r(x):P[W(x)>l|y]=r(x),x\in A\}$$ (5)

with *l* being the prevalence threshold; *W*(*x*), the predicted prevalence, *W*(*x*) = *g*^−1^{*T*(*x*)} with *g*^−1^{∙}, the inverse link function in the GLGM; and *r*(*x*) the point-wise exceedance probability. This probability was depicted on the prediction grid with grid cells of 1 km × 1 km spatial resolution. Finally, to assess the compatibility of the chosen spatial correlation structure, (i.e. correct specification of nugget *τ*^2^, and scale factor, *ϕ*), a Monte Carlo procedure was used (6). The procedure is detailed in S2 Text.

All statistical procedures were conducted in R version 4.0.5 [42]. The statistical significance level was set at 5%.

## Results

### Descriptive statistics

**Fig 2:**
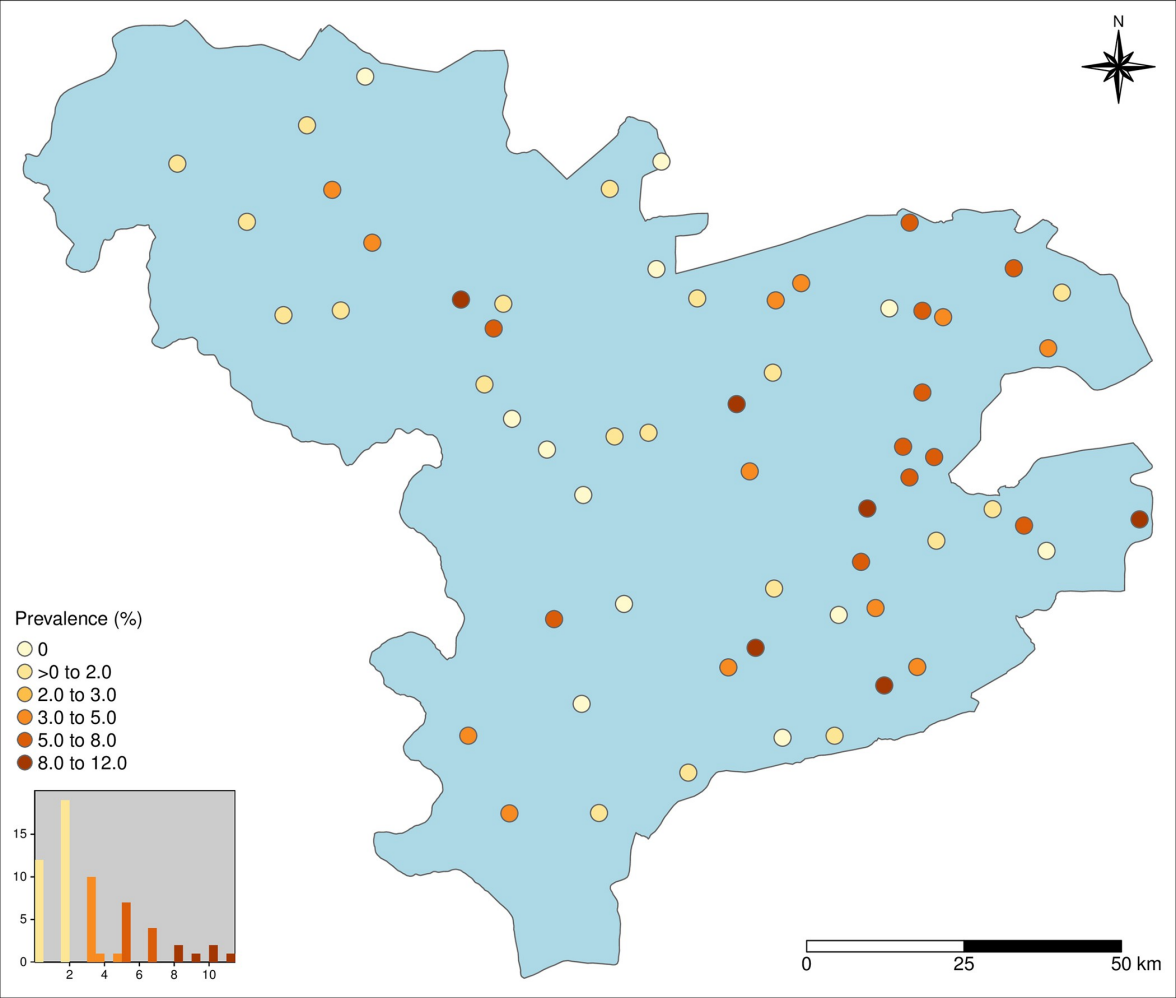
Study area with village-level data points and prevalence (derived from the individual-level dataset), and histogram of the prevalence (https://www.diva-gis.org/gdata).

Data were available for a total of 3598 individuals living in 60 villages in three provinces in Burkina Faso (Fig 2). The north-south dimension of the study area was 154 km, while its east-west dimension was 199 km, and its longest dimension was 195 km. Out of the 3598 individuals, 30 lacked information for the main outcome variable (i.e. Ag-ELISA result), 12 had no village name assigned, and 4 data points were co-located. Of the remaining 3551 participants, 115 (3.24%) tested positive in the Ag-ELISA for active hCC. The number of participants ranged between 52 and 66 per village, with a median of 59. Out of the 60 villages, 48 had positive cases, in those villages, the number of positive cases ranged from 1 to 7, with a median of 2. Overall, village prevalence estimates ranged from 0 to 11.5%, with a median of 1.85%. Villages with higher prevalence estimates seemed primarily located in the south-east of the study area (Fig 2).

**Fig 3:**
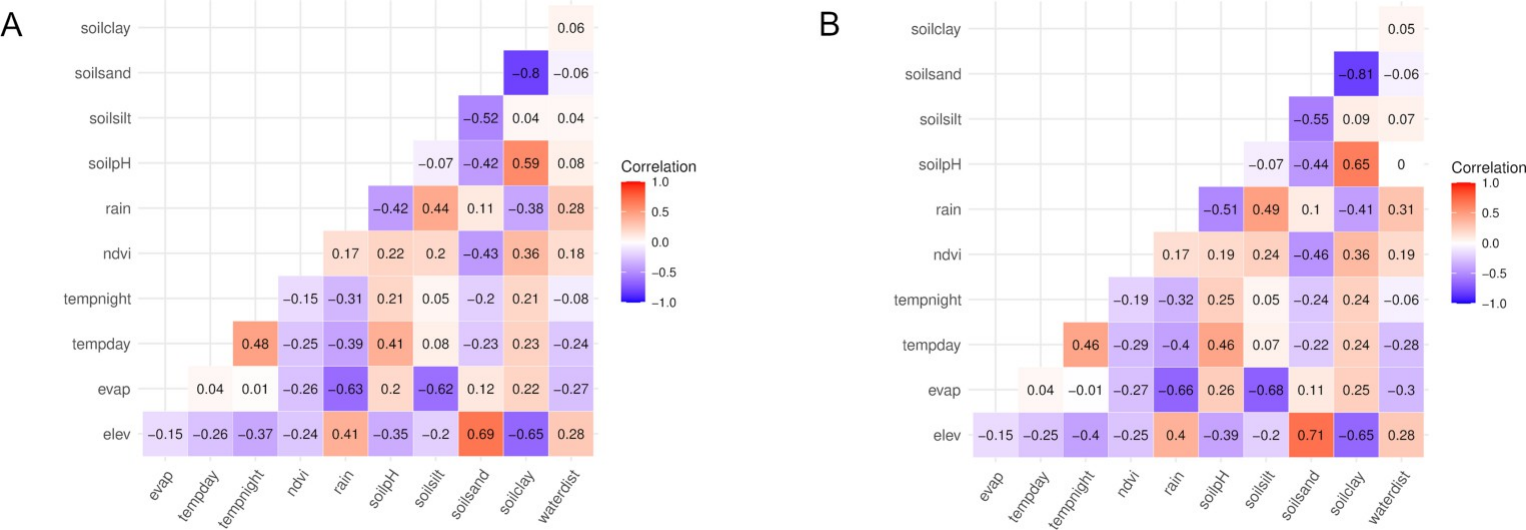
**Correlation plot for individual-level (A) and village-level (B) environmental data in the study area.** Evap: potential evapotranspiration, tempday: land surface temperature, day; tempnight: land surface temperature, night; ndvi: normalized difference vegetation index; rain: precipitation; soilpH: soil pH (0–5 cm); soilsilt: soil silt (0–5 cm); soilsand: soil sand (0-5cm); soilclay: soil clay (0–5 cm); waterdist: distance to the nearest river.

The environmental data showed considerable variation over the study area (S1 Fig and S2 Table), except for land cover, where type 31 (i.e. barren land) was predominant. For this reason, land cover was excluded from further analyses. A strong negative correlation existed between elevation and soil clay (individual-level data: Spearman *ρ* = -0.65), and between soil clay and soil sand (*ρ* = -0.80) (Fig 3A), whereas a positive correlation was observed between elevation and soil sand (*ρ* = 0.69). Correlations were similar for the village-level dataset (Fig 3B).

### Variogram

**Fig 4:**
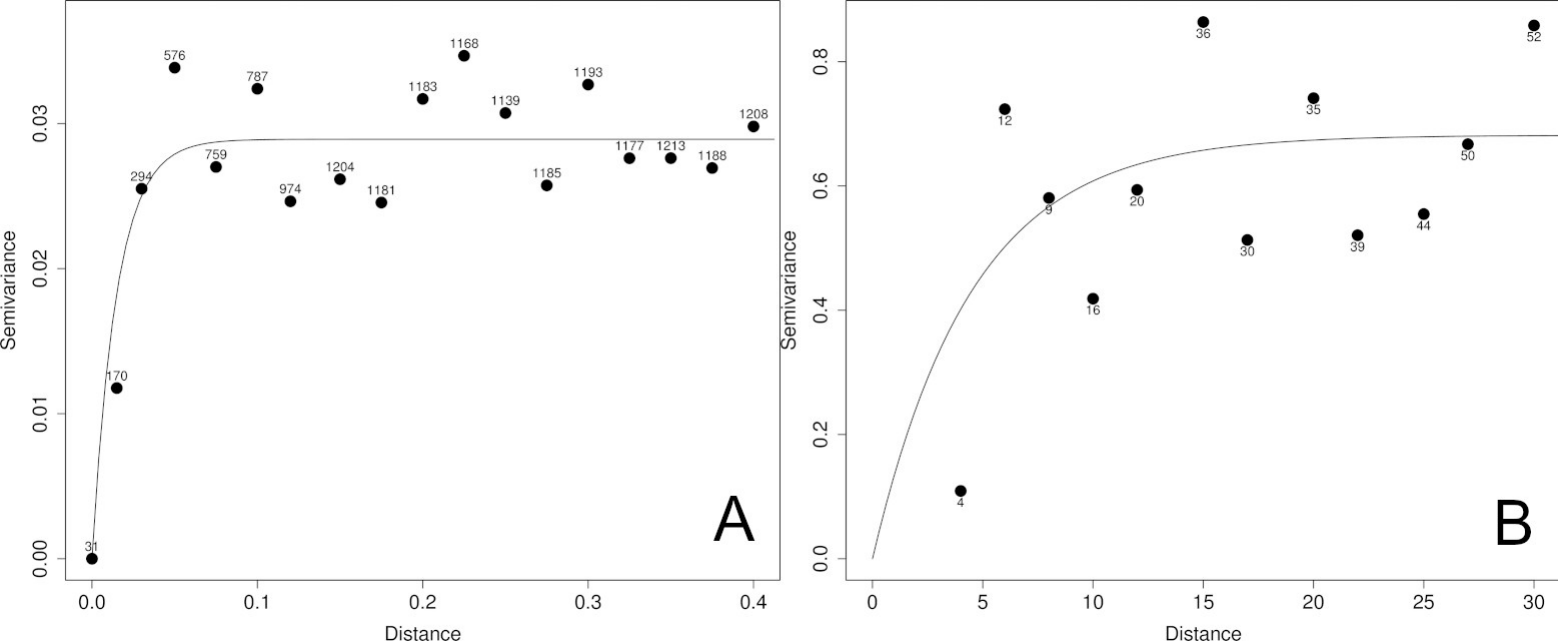
Final empirical variogram with fitted variogram model and number of pairs added for individual-level (A) and village-level (B) outcome data (distance in km).

Fig 4A illustrates the variogram for the individual-level data. When decreasing the maximum lag distance to 400 meters (18 bins, number of data pairs per bin: 31–1208), an increase in variogram values was observed when moving from zero to around 60 meter lag distance, suggesting spatial correlation at that scale. For the village-level data (Fig 4B), when the maximum lag distance was restricted to 30 km, one low variogram value was observed at the shortest lag distance for which the variogram was calculated, thereafter the values fluctuated around 0.60. The number of data pairs per bin (with 12 bins) varied between 4 and 52.

For the individual-level data, the sill, *σ*^2^, representing the variance of the spatial effect, was estimated at 0.029; the scale factor *ϕ* at 0.015 km with an estimated practical range, the distance over which data were spatially correlated, of 0.045 km. The nugget, *τ*^2^, representing sampling and/or measurement error, was estimated at 0. For the village-level data, the weighted least squares estimates for *σ*^2^, *ϕ*, and *τ*^2^ were 0.68, 4.50 km (estimated practical range: 13.5 km) and 0, respectively.

### Covariate selection

Based on the backward variable selection strategy, the final GLM included precipitation, distance to the nearest river and night land temperatures for the individual-level dataset while only precipitation and distance to the nearest river were retained for the village-level dataset (S3 Text).

### Geostatistical model

The fitted GLGM for the individual-level data, ℳ_1_, predicted that for each unit increase in precipitation (mm/month), the odds for a positive test result increased by 12% (exp(0.11) = 1.12 [95% confidence interval (CI): 1.11;1.12], 1.12–1 = 0.12), if the other covariates were kept constant (Table 2). Likewise, for each unit increase in distance to the nearest river (km), a decrease in the odds of 8% (exp(-0.088) = 0.92 [95%CI: 0.91;0.92], 1–0.92 = 0.08) and for each unit increase in night land temperature (°C), a decrease in the odds of 37% (exp(-0.47) = 0.63 [95%CI: 0.62;0.63], 1–0.63 = 0.37) were predicted, if the other covariates were kept constant. In the individual-level GLGM, ℳ_1_, and ℳ_3_, the variance of the spatial effect, *σ*^2^ was estimated at exp(-3.63) = 0.027 [95%CI: 0.025;0.028], whereas the shape factor, *ϕ* was estimated at exp(-4.21) = 0.015 [95%CI: 0.011;0.019]. The practical range, *u*, for hCC spatial correlation, after accounting for the covariates, was thus *u*≈3×*ϕ* = 0.045 km or 45 [95%CI: 34.3;57.8] meters. In other words, individual level data were estimated to be spatially correlated up to 45 meters.

**Table 2:** Parameter estimates in generalized linear geostatistical model for individual- and village-level data.

Model	Model-level	Parameter	Estimate	95%CI	*p*-value
ℳ_1_	Individual	Intercept	-9.54	[-10.0;-9.03]	<0.001
		Precipitation (mm/month)	0.11	[0.10;0.11]	<0.001
		Distance to the nearest river (km)	-0.088	[-0.090;-0.086]	<0.001
		Land temperature, night (°C)	-0.47	[-0.48;-0.46]	<0.001
		log(*σ*^2^)	-3.63	[-3.67;-3.58]	
		log(*ϕ*)	-4.21	[-4.47;-3.95]	
ℳ_2_	Village	Intercept	-20.4	[-33.4;-7.37]	<0.002
		Precipitation (mm/month)	0.10	[0.024;0.18]	0.011
		Distance to the nearest river (km)	-0.086	[-0.14;-0.035]	0.001
		log(*σ*^2^)	-1.19	[-1.62;-0.75]	
		log(*ϕ*)	2.24	[1.54;2.93]	
ℳ_3_	Individual	Intercept	-24.0	[-24.4;-23.6]	<0.001
		Precipitation (mm/month)	0.12	[0.12;0.13]	<0.001
		Distance to the nearest river (km)	-0.080	[-0.082;-0.078]	<0.001
		log(*σ*^2^)	-3.63	[-3.67;-3.58]	
		log(*ϕ*)	-4.21	[-4.47;-3.95]	

95%CI: 95% confidence interval

Similar effect sizes of the environmental variables were observed using the village-level dataset. Indeed, the fitted GLGM, ℳ_2_, predicted that for each unit increase in precipitation (mm/month), the odds for a positive test result was increased by 11% (exp(0.10) = 1.11 [95%CI: 1.02;1.20], 1.11–1 = 0.11), if the distance to the nearest river was kept constant. Moreover, for each unit increase in distance to the nearest river (km), a decrease in the odds for a positive test result by 8% (exp(-0.086) = 0.92 [95%CI: 0.87;0.97], 1–0.92 = 0.08) was predicted, if precipitation was kept constant. The variance of the spatial effect, *σ*^2^ was estimated at exp(-1.19) = 0.30 [95%CI: 0.20;0.47], whereas the shape factor, *ϕ* was estimated at exp(2.24) = 9.39 [95%CI: 4.66;18.7]. The practical range, *u*, for hCC spatial correlation was thus *u*≈3×*ϕ* = 28.2 [95%CI: 14.0;56.2] km, or in other words, the village-level data were estimated to be spatially correlated up to 28.2 km. No overfitting was observed for any of the GLGM (all |*ρ*_*P*_| < 0.35).

### Prediction

**Fig 5:**
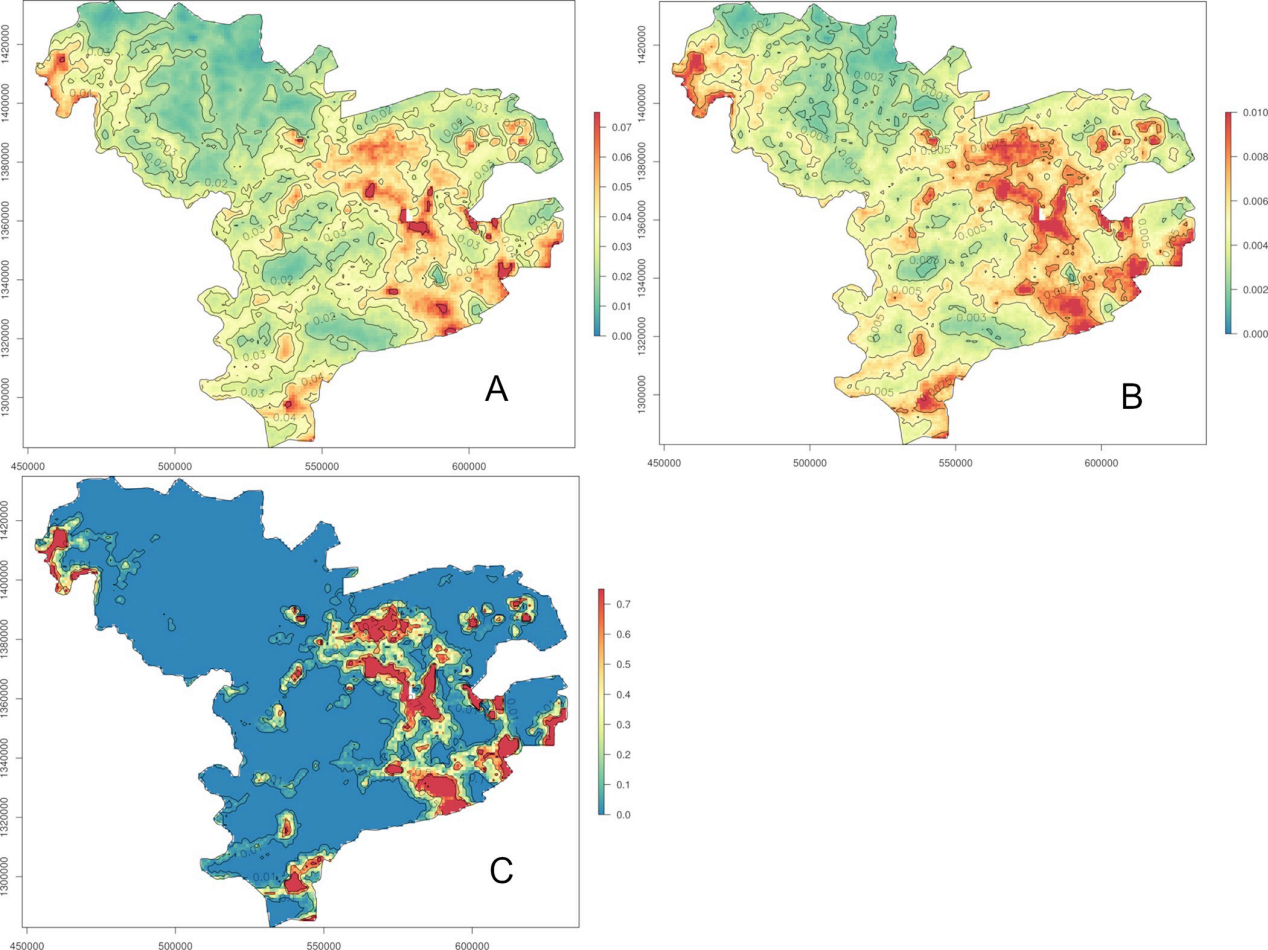
Predicted prevalence (A), standard errors (B) and probability to exceed 5% prevalence (C) based on the geostatistical model ℳ_1_ for the participant-level data (https://www.diva-gis.org/gdata).

**Fig 6:**
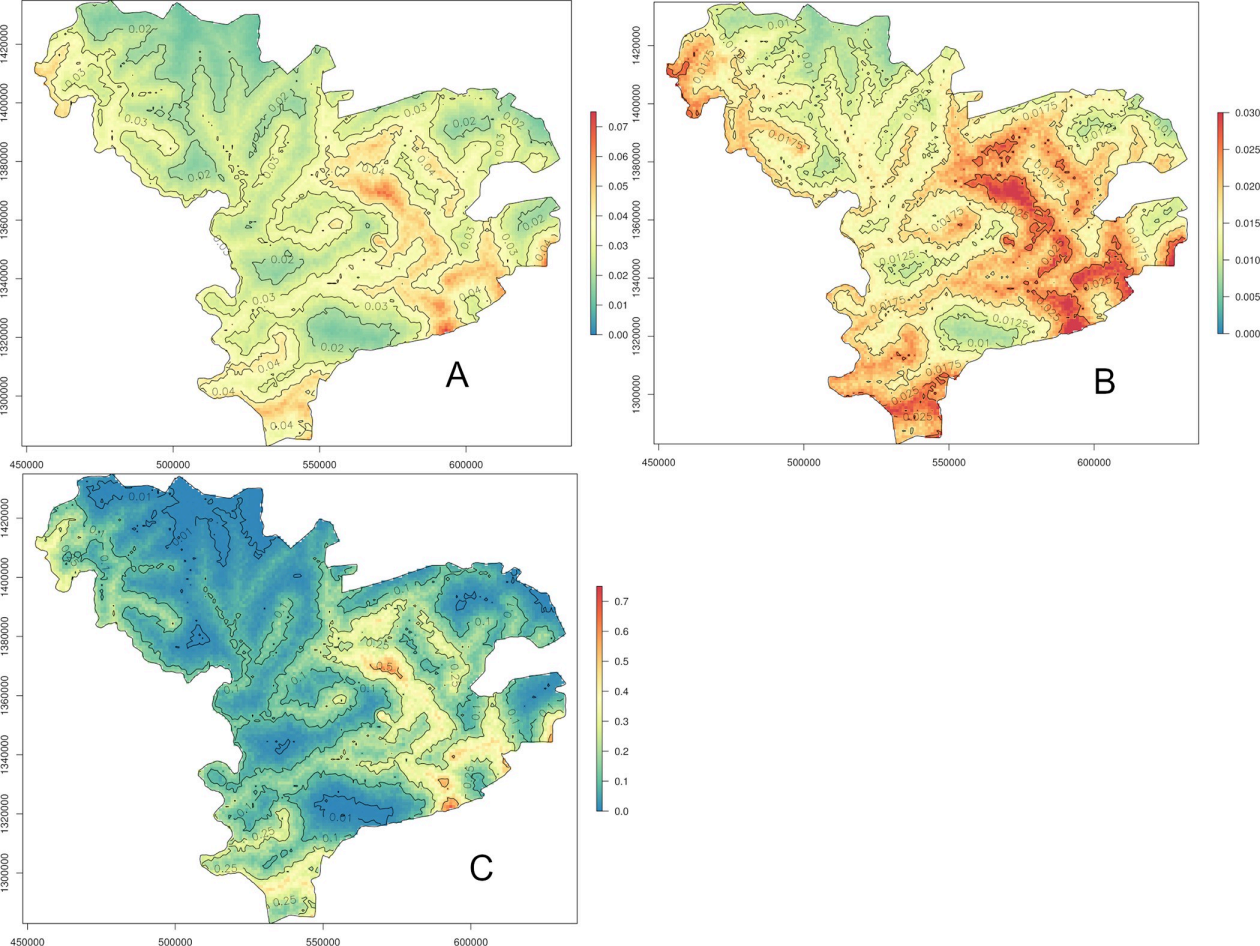
Predicted prevalence (A), standard errors (B) and probability to exceed 5% prevalence (C) based on the geostatistical model ℳ_2_ for the village-level data (https://www.diva-gis.org/gdata).

**Fig 7:**
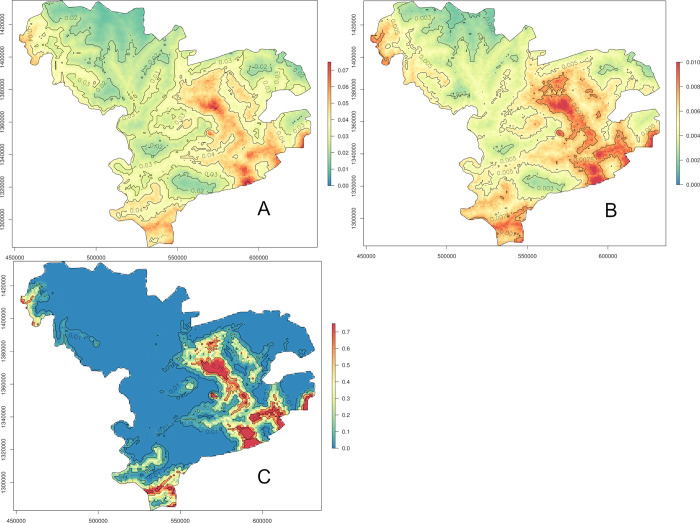
Predicted prevalence (A), standard errors (B) and probability to exceed 5% prevalence (C) based on the geostatistical model ℳ_3_ for the participant-level data (https://www.diva-gis.org/gdata).

Maps were drawn for the predicted hCC prevalence, standard error and probability of exceeding the 5% threshold (Figs 5–7). The dataset for night land temperatures had six missing values, resulting in a blank empty pixel at six locations on the prediction maps based on the individual-level GLGM ℳ_1_ (Fig 5A). The prediction maps for active hCC prevalence generated from the individual- and village-level GLGM, ℳ_1_, ℳ_2_ and ℳ_3_ (Figs 5A, 6A, 7A, respectively), identified important areas with hCC prevalence estimates of at least 4% in the south-east, as well as, albeit to a lesser extent, in the extreme south, and north-west of the study area, while patches of prevalence estimates below 2% were mainly apparent in the north and west.

The standard errors for the prevalence estimates exhibited a similar pattern as the predicted prevalences for all GLGM (Figs 5B, 6B, 7B), with higher standard errors in areas with higher prevalence estimates. In the maps based on the individual-level GLGM, ℳ_1_ and ℳ_3_, the standard errors were distinctly lower, as compared to those found for the village-level GLGM, ℳ_2_.

The map of the probability for the active hCC prevalence to exceed 5% (Figs 5C, 6C, 7C) confirmed the conclusions drawn from the predicted prevalence maps: the exceedance probability was highest in the south-east and north-west of the study area (patches of >75% probability). However, the village-level GLGM (ℳ_2_) produced a markedly smoother map of the exceedance probability as compared to the individual-level GLGM (ℳ_1_, and ℳ_3_) maps. In the village-level GLGM (ℳ_2_) map, patches of >25% probability could also be observed in the south-east and north-west of the study area. Moreover, in large parts of the remaining areas in the north, west and far east of the study area, the exceedance probability was consistently lower than 10%.

## Discussion

This study aimed to conduct a geostatistical analysis for active hCC in Burkina Faso, an endemic country in Sub-Saharan Africa. This analysis allows for the spatial prediction of the prevalence of active hCC at unsampled locations, which is informative to identify those areas that would benefit most from targeted intervention programmes. Indeed, the analysis resulted in a high resolution map (1 km × 1 km) of the predicted distribution of active hCC in the study area, the provinces Boulkiemdé, Sanguié, and Nayala, with important clusters of hCC in the south-east, as well as in the extreme south, and north-west. Moreover, the analysis indicated that the practical range for spatial correlation of survey values was very short for the individual-level dataset (45 meters), while rather large for the village-level dataset (28.2 km). The difference between these two distances could be due to the spatial aggregation of the village-level dataset (i.e. by design, all villages were at least 6 km apart from each other). Overall, these distances are a lot shorter than the 140 km for the municipality-level data observed by Galipó et al. [29]. However, a positive test result in the diagnostic test in that study (Ab-ELISA) indicates exposure to the parasite, whereas in our study (Ag-ELISA), it indicates active or current infection. Indeed, the Ab-ELISA can detect both viable, degenerating and calcified cysticerci, but also past exposure to the parasite that didn’t result in infection, and a resolved infection [35]. The Ag-ELISA on the other hand will only detect viable and early-stage degenerating cysticerci, as only those will excrete/secrete antigens [35]. Furthermore, the actual test performance of the antibody-ELISA run on dried blood spots, as used in the Galipó study [29] is unclear considering that the paper describing the method does not clearly justify or explain study groups [43]. Moreover, the study of Galipó et al. [29] was a national survey, sampling 133 out of 1122 municipalities, in 23 out of 32 of the country’s departments, whereas we sampled all departments in three provinces. Overall, it is therefore not clear to what extent the observed ranges can be compared.

The results of our fitted geostatistical models demonstrated that increasing precipitation, and decreasing distance to the nearest river were associated with an increased probability for a positive test result, and thus the presence of active hCC. In the individual-level model, decreasing night land temperatures were additionally found to be associated with increased probability for a positive test result. These results are in line with earlier studies indicating that *Taenia* spp. eggs survive better in a moist environment, while high temperatures are detrimental for their survival [36]. As expected, the variability in the hCC prediction maps closely resembled those in the maps of the selected covariates. Moreover, for the individual-level GLGM, the standard errors of the predicted prevalences were markedly lower than those generated by the village-level GLGM, partly explained by the lack of high resolution data for the latter as compared to the former. Finally, the exceedance probability map generated based on the village-level GLGM was considerably smoother than those for the individual-level GLGM, which could be explained by the combined effect of lower data density, and the smoothness in the environmental data and in the the spatial structure adopted for the village-level GLGM.

The analysis also highlighted several challenges related to the use of the survey data on hCC for prediction mapping. For the individual-level modelling, a large (ungrouped) dataset was used (*n* = 3551), the analysis of which was associated with a relatively high computational burden. For the village-level modelling on the other hand, the number of data points was suboptimal (*n* = 60). Webster and Oliver [44] had pointed out that at least 100 observations are necessary to ensure reliable calculation of the variogram, while Journal and Huijbregts [45] have indicated that at least 30 to 50 data-pairs are necessary for each distance bin. In the current study, the 60 data points resulted in a low number of data-pairs available for the calculation of the empirical variogram for the village-level modelling, especially at the small distances, affecting the reliability of the variogram model parameter estimation. Moreover, the sampling design dictated that villages needed be at least 5 km apart to be included, thus hampering the investigation of spatial continuity at the short distances. For both analyses, the variogram analysis estimated the nugget, representing sampling and/or measurement error (background noise) at 0. In practice, however, the absence of a nugget seems unrealistic, as sampling and/or measurement error might be present due to several factors.

Furthermore, a stratified multi-level random sampling approach was chosen for the purpose of a cluster-randomized trial to be conducted in the study area, but it is not necessarily ideal for conducting a geostatistical analysis. At the two extremes of sampling designs for geostatistical modelling, are completely random designs (where point samples are collected randomly across the study area), relatively efficient for estimation purposes; and completely regular designs (where point samples are, for instance, collected at regular distances across the study area) for efficient spatial prediction [46]. The latter however assumes that the variogram model parameters are known, which is most often not the case (these parameters then need to be estimated from a single dataset), and for most geostatistical problems, a compromise is needed between both goals [6,47]. This was also the case in the present work, where the variogram model parameters were estimated from the same datasets used for prediction. It has been shown that the completely regular design with close pairs added is one of the most effective designs in this case (e.g., Diggle and Lophaven [48]), which is recommended for future geostatistical analyses for hCC.

A number of limitations with regard to the interpretation and validity of study results were also present. There were uncertainties related to the measurement of the outcome variable. Firstly, the applied diagnostic tool is not a perfect test, its performance is characterised by a sensitivity of 90% and specificity of 98%, thus impacting the estimation of the true prevalence [35]. Furthermore, the selection of participants was not random, rather they were selected from the concessions depending on pig type (i.e. sows, or piglets, although the levels of the variable were not mutually exclusive). The presence of pigs could be linked to presence of *T*. *solium* carriers and hence to the presence of hCC cases. In most villages, this should not have been a huge issue since most concessions actually had pigs, so sampling bias due to this factor was assumed to be minimal. It is also important to note that the survey data were considered as point data throughout the analysis, i.e. it was assumed that the spatial support of the observations was negligible compared to the total size of the study area, and implicitly also that there was no uncertainty in the location of these point data. However, for the data at hand, some reservations exist about the extent to which this assumption holds. Participants did not reside at a single point location only, but moved over a certain surface in space (spatial support). This surface was unknown and, moreover, differed in size and shape between different participants. For both datasets, this had an impact on the calculation of variogram values at short distances. For actual point data, the minimum distance for which a variogram value can be calculated is limited to the smallest sampling distance. For point data that are actually surface data, such as the data at hand, there can be an additional restriction due to the minimum area (size) represented in the data points [49].

Several simplifications and assumptions were also made, which could have impacted the quality of the environmental data used in the analysis. First, either yearly or mid-year datasets were often the only ones available, which resulted in differences in temporal support across the environmental variables. These choices also resulted in the assumption that there was no considerable temporal variation, or that the observation in the middle of the sampling period was sufficiently representative of the entire sampling period. Additionally, there was also a mismatch between time of measurement (i.e. temporal support) of the explanatory variables and the outcome data which came from a cross-sectional study conducted between February 2011 and January 2012. For the village-level dataset, the mean value of the sampled survey points was used as village-level value for each environmental variable, resulting in loss of within-village variability and an assumption that the mean was a representative summary statistic. For the soil variables, it was assumed that the top layer (0–5 cm depth) was the most relevant layer for transmission of *T*. *solium*. However, it is not inconceivable that eggs of the parasite can survive at larger depths, and that in case of erosion due to heavy rainfall, especially in combination with a considerable slope, these deeper layers could also contribute to the transmission. Finally, the analysis combined environmental and outcome values for surveys points in three provinces in Burkina Faso, thus assuming the structure of the spatial processes is similar in the three provinces. This could be further investigated in separate analyses for the three provinces. Overall, however, this study remains the only study of its type, and will guide further studies in the field.

## Supporting information

S1 STROBE Checklist (DOCX)

S1 Text Covariate selection procedure: methodology. (DOCX)

S2 Text Validation procedure. (DOCX)

S3 Text Covariate selection procedure: results. (DOCX)

S4 Text Validation results. (DOCX)

S1 Table Detailed data sources for the environmental data considered as potential explanatory variables for the spatial distribution of *T. solium*. (DOCX)

S2 Table Summary measures for environmental data extracted for individual- and village-level datasets. (DOCX)

S3 Table Fitted generalized linear models for the individual-level and village-level datasets. (DOCX)

S4 Table Fitted generalized linear mixed models for the individual-level and village-level datasets. (DOCX)

S1 Fig Maps of the environmental variables at the study area. (DOCX)

S2 Fig Relationship between village-level environmental variables and empirical logit outcome (Part A). (Green = linear regression, blue = general additive model with penalized smoother).
(DOCX)

S3 Fig Relationship between village-level environmental variables and empirical logit outcome (Part B). (Green = linear regression, blue = general additive model with penalized smoother).
(DOCX)

S4 Fig Relationship between individual-level environmental variables and outcome (Part A). (Green = linear regression, blue = general additive model with penalized smoother).
(DOCX)

S5 Fig Relationship between individual-level environmental variables and outcome (Part B). (Green = linear regression, blue = general additive model with penalized smoother).
(DOCX)

S6 Fig Testing for residual spatial correlation for the village-level data (distance in km). (DOCX)

S7 Fig Test the fit of the final geostatistical model for the village-level data (distance in km). (DOCX)

S1 Data Village data. (XLSX)
