# Supplementary material for: Geostatistical analysis of active human cysticercosis: Results of a large-scale study in 60 villages in Burkina Faso
Source: PLoS Negl Trop Dis. 2023 Jul 26;17(7):e0011437. doi: 10.1371/journal.pntd.0011437 (PMC10370738; doi:10.1371/journal.pntd.0011437)
Supplement: S1 STROBE Checklist — (DOCX) [file pntd.0011437.s001.docx]

**S1 STROBE Checklist**

STROBE Statement—checklist of items that should be included in reports of observational studies

|  | Item No | Recommendation | Page No. | Relevant text from manuscript |
| --- | --- | --- | --- | --- |
| **Title and abstract** | 1 | *(*a) Indicate the study’s design with a commonly used term in the title or the abstract | 2 | Mentioned in abstract |
|  |  | (*b*) Provide in the abstract an informative and balanced summary of what was done and what was found | 2 | Done |
| Introduction | | |  |  |
| Background/rationale | 2 | Explain the scientific background and rationale for the investigation being reported | 4-5 | Done |
| Objectives | 3 | State specific objectives, including any prespecified hypotheses | 5 | Main objective mentioned in last paragraph of introduction. |
| Methods | | |  |  |
| Study design | 4 | Present key elements of study design early in the paper | 5 | Mentioned in “Study design” section. |
| Setting | 5 | Describe the setting, locations, and relevant dates, including periods of recruitment, exposure, follow-up, and data collection | 5-6 | Mentioned in “Study design” |
| Participants | 6 | (*a*) *Cross-sectional study*—Give the eligibility criteria, and the sources and methods of selection of participants | 5-7 | Described in “Study design” and “Participants” |
| Variables | 7 | Clearly define all outcomes, exposures, predictors, potential confounders, and effect modifiers. Give diagnostic criteria, if applicable | 7-9 | Described in “Outcome data” and “Environmental data” sections |
| Data sources/ measurement | 8* | For each variable of interest, give sources of data and details of methods of assessment (measurement). Describe comparability of assessment methods if there is more than one group | 7-9 | See Item (7) |
| Bias | 9 | Describe any efforts to address potential sources of bias | 9-12 | Previously used methods were applied to measure variables of interest. |
| Study size | 10 | Explain how the study size was arrived at | 5-7 | See item (6). Study size was based on the effectiveness of the randomized community trial based on expected follow-up data. |
| Quantitative variables | 11 | Explain how quantitative variables were handled in the analyses. If applicable, describe which groupings were chosen and why | 7-12 | All variables were included. |
| Statistical methods | 12 | *(*a) Describe all statistical methods, including those used to control for confounding | 9-12 | See “Data analysis” and the supplemental files. Multivariable analyses were used to measure confounding. |
|  |  | (*b*) Describe any methods used to examine subgroups and interactions |  | NA |
|  |  | (*c*) Explain how missing data were addressed | Results | Description of how missing data were addressed are described in the first paragraph of the results. |
|  |  | (*d*) *Cross-sectional study*—If applicable, describe analytical methods taking account of sampling strategy | 9 | Models were run on two databases: one at the individual-level, and another one at the village-level. Model results were compared afterwards. |
|  |  | (*e*) Describe any sensitivity analyses | NA | NA |

Continued on next page

| Results | | | Page No. | Relevant text from manuscript |
| --- | --- | --- | --- | --- |
| Participants | 13* | (a) Report numbers of individuals at each stage of study—eg numbers potentially eligible, examined for eligibility, confirmed eligible, included in the study, completing follow-up, and analysed | Carabin *et al.* 2015 |  |
|  |  | (b) Give reasons for non-participation at each stage |  | There was minimal refusal to participate |
|  |  | (c) Consider use of a flow diagram | Carabin *et al.* 2015 |  |
| Descriptive data | 14* | (a) Give characteristics of study participants (eg demographic, clinical, social) and information on exposures and potential confounders | Carabin *et al.* 2015 |  |
|  |  | (b) Indicate number of participants with missing data for each variable of interest | 12 | Done |
| Outcome data | 15* | *Cross-sectional study—*Report numbers of outcome events or summary measures | 12 | Done |
| Main results | 16 | *(*a) Give unadjusted estimates and, if applicable, confounder-adjusted estimates and their precision (eg, 95% confidence interval). Make clear which confounders were adjusted for and why they were included | 12-21 | Results |
|  |  | (*b*) Report category boundaries when continuous variables were categorized | NA | NA |
|  |  | (*c*) If relevant, consider translating estimates of relative risk into absolute risk for a meaningful time period | NA | NA |
| Other analyses | 17 | Report other analyses done—eg analyses of subgroups and interactions, and sensitivity analyses | 12-21 | Geostatistical model results. |
| Discussion | | |  |  |
| Key results | 18 | Summarise key results with reference to study objectives | 22-23 | Done |
| Limitations | 19 | Discuss limitations of the study, taking into account sources of potential bias or imprecision. Discuss both direction and magnitude of any potential bias | 23-25 | Limitations were discussed. |
| Interpretation | 20 | Give a cautious overall interpretation of results considering objectives, limitations, multiplicity of analyses, results from similar studies, and other relevant evidence | 22-25 | Done |
| Generalisability | 21 | Discuss the generalisability (external validity) of the study results | 25 | Discussion |
| Other information | | |  |  |
| Funding | 22 | Give the source of funding and the role of the funders for the present study and, if applicable, for the original study on which the present article is based | 25 | See “Funding information”. |

*Give information separately for cases and controls in case-control studies and, if applicable, for exposed and unexposed groups in cohort and cross-sectional studies.

**Note:** An Explanation and Elaboration article discusses each checklist item and gives methodological background and published examples of transparent reporting. The STROBE checklist is best used in conjunction with this article (freely available on the Web sites of PLoS Medicine at http://www.plosmedicine.org/, Annals of Internal Medicine at http://www.annals.org/, and Epidemiology at http://www.epidem.com/). Information on the STROBE Initiative is available at [www.strobe-statement.org](http://www.strobe-statement.org).

Carabin H, Millogo A, Cissé A, Gabriël S, Sahlu I, Dorny P, Bauer C, Tarnagda Z, Cowan LD, Ganaba R. Prevalence of and factors associated with human cysticercosis in 60 Villages in three provinces of Burkina Faso. PLoS Negl Trop Dis. 2015;9:1–20. doi: 10.1371/journal.pntd.0004248.
