# Supplementary material for: Geostatistical analysis of active human cysticercosis: Results of a large-scale study in 60 villages in Burkina Faso
Source: PLoS Negl Trop Dis. 2023 Jul 26;17(7):e0011437. doi: 10.1371/journal.pntd.0011437 (PMC10370738; doi:10.1371/journal.pntd.0011437)
Supplement: S1 Text — (DOCX) [file pntd.0011437.s002.docx]

**S1 Text: Covariate selection procedure: methodology**

Scatter plots were drawn to investigate the relationship between the outcome variable or empirical logit of the outcome, for the individual-level and village-level data respectively, and each environmental variable. Both a linear fit and a fit of a generalized additive model (GAM) (1) with a penalized smoother were added to the graph.

Additionally, generalized linear models (GLM) were run of the general form:

|  | $log\left\{ \frac{p(x_{i})}{1-p\left( x_{i} \right)} \right\}=d\left( x_{i} \right)^{t}\beta$ | (S1) |
| --- | --- | --- |

with $p(x_{i})$ being the probability for a positive test result; and $d\left( x_{i} \right)^{t}\beta$, the transposed column vector of exploratory variables $d_{i}$ at point $x_{i}$with regression coefficients $\beta$.

A backward stepwise selection approach was used to find the most parsimonious GLM between outcome and environmental data. Models were compared using the likelihood-ratio test (LRT), and multicollinearity was checked for by exploring the Pearson’s $\rho_{p}$ correlation coefficients between regression coefficients and by calculation of the variance inflation factor (VIF). An absolute value for the correlation coefficient above 0.70 and VIF above 5 were considered indicative for multicollinearity, respectively (2).

Next, the presence of residual spatial correlation was investigated using a Monte Carlo method (3). The final set of covariates as chosen in the backward stepwise selection approach for the GLM (Equation S1) was first included in a generalized linear mixed model (GLMM):

|  | $log\left\{ \frac{p(x_{i})}{1-p\left( x_{i} \right)} \right\}=d\left( x_{i} \right)^{t}\beta+Z_{i}$ | (S2) |
| --- | --- | --- |

with $p(x_{i})$ being the probability for a positive test result; $d\left( x_{i} \right)^{t}\beta$, the transposed column vector of the exploratory variables $d_{i}$ at point $x_{i}$with regression coefficients $\beta$; and $Z_{i}$, as independent random effects (participant or village, depending on the database) with $Z_{i}\sim N(0,\tau^{2})$.

An empirical variogram was then drawn up for the predicted residuals, $Z_{i}$, using the formula:

|  | $\hat{V}_{0}\left( u \right)=\frac{1}{2\left\vert N(u) \right\vert}\sum_{\left( h,k \right)\in N(u)} (\hat{Z}_{h}-\hat{Z}_{k})^{2}$ | (S3) |
| --- | --- | --- |

with $N(u)$ being the number of data-pairs at distance $u$ apart from each other; and with $\hat{Z}_{h}$ and $\hat{Z}_{k}$, the residuals for the outcome variable at location $h$ and $k$, respectively. Data pairs were first classified in distance bins and the empirical variogram was averaged for each bin.

Then, the empirical variogram of the $\hat{Z}_{i}$ was calculated by randomly permuting the labelling of the $\hat{Z}_{i}$ at the fixed locations $x_{i}$. The process was repeated 10 000 times, and the 95% probability interval of variogram values under the assumption of spatial independence, was calculated. In case the calculated variogram, $\hat{V}_{0}\left( u \right)$, lay outside the 95% probability interval, there was evidence for residual spatial correlation.

The following test statistic was subsequently used to investigate the hypothesis of spatial independence (3):

|  | $T =\sum_{k=1}^{K} \left\vert N\left( u_{k} \right) \right\vert\left[ \hat{V}\left( u_{k} \right)-\tau^{2} \right]^{2}$ | (S4) |
| --- | --- | --- |

where $K$ is the number of bins; $N(u_{k})$ is number of data-pairs at distance $u$ apart from each other for bin $k$; $\hat{V}\left( u_{k} \right)$ the variogram for the predicted residuals for distance bin $k$; and $\tau^{2}$ the variance of the random effects, $Z_{i}$, included in the GLMM (Equation S2). The $p$-value was then calculated as the proportion of $T$-values for the permuted $\hat{Z}_{i}$ larger than the $T$-value for the orignal $\hat{Z}_{i}$.

**References**

1. Hastie T, Tibshirani R. Generalized Additive Models. London: Chapman and Hall; 1990.
2. Sheather S. A modern approach to regression with R. New York, NY: Springer; 2009.
3. Diggle PJ, Giorgi E. Model-based Geostatistics for Global Public Health. Methods and Applications. Boca Raton, Florida: CRC Press, Chapman & Hall; 2019.
