# Supplementary material for: Geostatistical analysis of active human cysticercosis: Results of a large-scale study in 60 villages in Burkina Faso
Source: PLoS Negl Trop Dis. 2023 Jul 26;17(7):e0011437. doi: 10.1371/journal.pntd.0011437 (PMC10370738; doi:10.1371/journal.pntd.0011437)
Supplement: S2 Text — (DOCX) [file pntd.0011437.s003.docx]

**S2 Text: Validation procedure**

In order to validate the fit of the chosen spatial correlation structure (i.e. correct specification of nugget $\tau^{2}$, and scale factor, $\phi$), a Monte Carlo procedure was used (Diggle and Giorgi, 2019)[1]. Similarly to the test for residual spatial correlation, the procedure started with the fitted GLMM that included the final set of covariates as chosen in the backward stepwise selection approach for the GLM, to allow for estimation $Z_{i}$ (Equation S2), and the calculation of the variogram, $\hat{V}_{0}\left( u \right)$, for the estimates of $Z_{i}$ (Equation S3).

Next, the model parameters in fitted GLGM (Equation 3 in the main text) were fixed at their maximum likelihood estimates, and a high number, of binomial datasets (10 000) were then generated with simulation of $S(x_{i})$, $Z_{i}$ and $Y_{i}$ as realisation of $d\left( x_{i} \right)^{t}\beta+S\left( x_{i} \right)+Z_{i}$. For each dataset, $Z_{i}$ values were estimated using the same GLMM as described in Equation S2, based on the simulated outcome values $Y_{i}$. The variogram for these $Z_{i}$ was calculated and the 95% probability interval of variogram values was constructed for each distance bin. In case the variogram generated based on the GLMM of the original data, $\hat{V}_{0}\left( u \right)$, fell within the 95% probability envelope, this was pointing to the correct spatial correlation structure.

Finally, a goodness-of-fit test was run, with calculation of the following test statistic (Diggle and Giorgi, 2019)[1]:

|  | $T =\sum_{k=1}^{K} \left\vert N\left( u_{k} \right) \right\vert\left[ \hat{V}\left( u_{k} \right)-V\left( u_{k};\phi\right) \right]^{2}$ | (S5) |
| --- | --- | --- |

where $K$ is the number of bins; $N(u_{k})$ is number of data-pairs at lag distance $u$ apart from each other for bin $k$; $\hat{V}\left( u_{k} \right)$ the empirical variogram for the predicted residuals for the distance bin $k$; and $V\left( u_{k};\phi\right)$ the fitted variogram model for the predicted residuals for the distance bin $k$. The $p$-value was then calculated as the proportion of $T$-values for the simulated $\hat{Z}_{i}$ larger than the $T$-value for the original $\hat{Z}_{i}$.

**References**

1. Diggle PJ, Giorgi E. Model-based Geostatistics for Global Public Health. Methods and Applications. Boca Raton, Florida: CRC Press, Chapman & Hall; 2019.
