# Supplementary material for: Geostatistical analysis of active human cysticercosis: Results of a large-scale study in 60 villages in Burkina Faso
Source: PLoS Negl Trop Dis. 2023 Jul 26;17(7):e0011437. doi: 10.1371/journal.pntd.0011437 (PMC10370738; doi:10.1371/journal.pntd.0011437)
Supplement: S3 Text — (DOCX) [file pntd.0011437.s004.docx]

**S3 Text: Covariate selection procedure: results**

For most environmental variables in the village-level dataset, the relationship with the empirical logit of the outcome was rather noisy (S2-S3 Figs). An increasing linear relationship was observed for elevation, precipitation and soil sand, whereas a decreasing linear relationship for evaporation, NDVI, distance to water, soil pH and soil silt. For day and night land temperatures, a rather curvilinear decreasing relationship could be observed. Soil clay, finally, exhibited a non-linear relationship with the empirical logit. For the individual-level dataset, the interpretation of the same figures was less straight forward (S4-S5 Figs).

Next, the most parsimonious generalized linear model (GLM) model was sought for both for the individual-level and village-level outcome data. The process was started with a model containing all environmental variables. Due to the strong correlation between the coefficients for soil pH, soil sand and soil clay (all $\left| \rho_{p} \right|$ > 0.80 in the individual-level model) and the high variance inflation factor (VIF) for the four soil variables (all VIF > 5), it was opted to retain only one soil variable in the model. Soil clay was chosen as it is considered most informative concerning soil moisture content and occurrence of biogeochemical processes. The backward stepwise selection approach was then applied to find the most parsimonious model, with models being compared by likelihood ratio testing (LRT). The fitted GLM for the individual-level data, $\mathcal{M}_{S1}$, retained precipitation, distance to the nearest river and night land temperatures as explanatory variables, while precipitation and distance to the nearest river for the village-level GLM, $\mathcal{M}_{S2}$ (S3 Table). For comparative purposes of the final prediction maps generated by the village- and individual-level data, another GLM was fitted to the individual-level, $\mathcal{M}_{S3},$ which only included precipitation and distance to the nearest river.

The fitted GLM for the individual-level data, $\mathcal{M}_{S1}$, predicted for each unit increase in precipitation (mm/month), an increase in the odds for a positive test result by 12% (exp(0.11) = 1.12 [95%CI: 1.03;1.20], 1.12 – 1 = 0.12), if the other covariates were kept constant. Likewise, for each unit increase in distance to the nearest river (km), a decrease in the odds by 8% (exp(-0.085) = 0.92 [95%CI: 0.85;0.98], 1 - 0.92 = 0.08) and for each unit increase in night land temperature (°C), a decrease in the odds by 37% was predicted (exp(-0.47) = 0.63 [95%CI: 0.43;0.91], 1 - 0.63 = 0.37), if the other covariates were kept constant. For the village-level data, the fitted model, $\mathcal{M}_{S2},$ predicted for each unit increase in precipitation, an increase in the odds for a positive test result by 13% (exp(0.12) = 1.13 [95%CI: 1.05;1.23], 1.13 – 1 = 0.13), and for each unit increase in distance to the nearest river (km), a decrease in the odds for a positive test result by 8% (exp(-0.085) = 0.92 [95%CI: 0.85;0.99], 1 – 0.92) was predicted, if the other covariates were kept constant respectively. The GLM fitted with the individual-level data, including the same covariates, $\mathcal{M}_{S3}$, had similar estimates as the latter.

To test for residual spatial correlation, a GLMM was fitted first with the explanatory variables included in models $\mathcal{M}_{S1}$, $\mathcal{M}_{S2}$, $\mathcal{M}_{S3}$. The same estimates for the coefficients were found for the models $\mathcal{M}_{S4}$, $\mathcal{M}_{S5}$, $\mathcal{M}_{S6}$ as for the models $\mathcal{M}_{S1}$, $\mathcal{M}_{S2}$, $\mathcal{M}_{S3}$, respectively, although for the village-level GLMM ($\mathcal{M}_{S5})$, the intercept estimate and the 95% confidence intervals changed and the effect of distance to the nearest river was not significant anymore (S3 Table). For the individual-level GLMM ($\mathcal{M}_{S4}$, $\mathcal{M}_{S6}$), the variance of the random effect, $\tau^{2}$, was estimated at 0, whereas for the village-level GLMM ($\mathcal{M}_{S5})$ at 0.20 [95% confidence interval (CI): 0;0.76].

Then, the aim was to investigate the presence of residual spatial correlation by means of these GLMM. However, for the individual-level GLMM ($\mathcal{M}_{S4}$, $\mathcal{M}_{S6}$), the calculation of the empirical variogram for the residuals as well as of the 95% confidence interval for the randomly labelled residuals could not be run as the variance of the random effect, $\tau^{2}$, was estimated at 0. For the village-level GLMM ($\mathcal{M}_{S5}$), the variogram for the residuals had a similar pattern as for the empirical logit, with the typical rising trend in values with increasing distances (S6 Fig). Nevertheless, the empirical variogram did not exceed the 95% confidence interval for the variogram values of the randomly permuted residuals (a very wide 95% confidence interval was observed at the low distances). Moreover, the hypothesis of spatial independence could not be rejected ($p$= 0.673).
