# Supplementary material for: Geostatistical analysis of active human cysticercosis: Results of a large-scale study in 60 villages in Burkina Faso
Source: PLoS Negl Trop Dis. 2023 Jul 26;17(7):e0011437. doi: 10.1371/journal.pntd.0011437 (PMC10370738; doi:10.1371/journal.pntd.0011437)
Supplement: S4 Text — (DOCX) [file pntd.0011437.s005.docx]

**S4 Text: Validation results**

The compatibility of the correlation structure incorporated in the GLGM $\mathcal{M}_{1}$, $\mathcal{M}_{2}$, and $\mathcal{M}_{3}$ for the data at hand was investigated by means of simulation and a statistical test. Again, for the individual-level GLGM $\mathcal{M}_{1}$ and $\mathcal{M}_{3}$, this could not investigated used the described methodology, as the variance of the random effect, $\tau^{2}$ in the GLMM, $\mathcal{M}_{S4}$ and $\mathcal{M}_{S6},$was estimated at 0. For the village-level model, $\mathcal{M}_{2}$, the empirical variogram of the estimated residuals of the GLMM lay within the 95% confidence interval of variogram values based on the simulated samples (S7 Fig). Moreover, the goodness-of-fit test was not significant. Therefore, there was lack of evidence against the fitted correlation function. Nevertheless, the confidence interval was rather wide, thus pointing to the lack of power of the test.
