# Supplementary material for: Geostatistical analysis of active human cysticercosis: Results of a large-scale study in 60 villages in Burkina Faso
Source: PLoS Negl Trop Dis. 2023 Jul 26;17(7):e0011437. doi: 10.1371/journal.pntd.0011437 (PMC10370738; doi:10.1371/journal.pntd.0011437)
Supplement: S1 Table — (DOCX) [file pntd.0011437.s006.docx]

**S1 Table. Detailed data sources for the environmental data considered as potential explanatory variables for the spatial distribution of *T. solium***

| Variable | Source | URL/citation |
| --- | --- | --- |
| Country/province boundary | DIVA-GIS | <http://www.diva-gis.org/gdata> |
| Potential evapotranspiration | CGIAR-CSI | 1 |
| Elevation | CGIAR SRTM | 2 |
| Land cover | MODIS-Terra | 3 |
| Land surface temperature day, night | MODIS-Terra | 4 |
| Normalized Difference Vegetation Index | MODIS-Terra | 5 |
| Precipitation | WorldClim | 6 |
| Water lines (to calculate Distance to river) | DIVA-GIS | <http://www.diva-gis.org/gdata> |
| Soil pH (0-5 cm) | ISRIC | 7 |
| Soil sand (0-5 cm) | ISRIC | 7 |
| Soil clay (0-5 cm) | ISRIC | 7 |
| Soil silt (0-5 cm) | ISRIC | 7 |

CGIAR-CSI: Consultative Group for International Agricultural Research - Consortium for Spatial Information (CGIAR-CSI); CGIAR SRTM: CGIAR Shuttle Radar Topography Mission; DIVA-GIS: Data-Interpolating Variational Analysis - Geographic Information System; ISRIC: International Soil Reference and Information Centre; MODIS: Moderate Resolution Imaging Spectroradiometer; NDVI: Normalized Difference Vegetation Index; WorldClim: World Climate

**Reference**

1. Trabucco, Antonio; Zomer, Robert (2019): Global Aridity Index and Potential Evapotranspiration (ET0) Climate Database v2. figshare. Dataset. <https://doi.org/10.6084/m9.figshare.7504448.v3>
2. Jarvis A., H.I. Reuter, A. Nelson, E. Guevara, 2008, Hole-filled seamless SRTM data V4, International Centre for Tropical Agriculture (CIAT), available from <https://srtm.csi.cgiar.org>.
3. Friedl, M., Sulla-Menashe, D. (2019). MCD12Q1 MODIS/Terra+Aqua Land Cover Type Yearly L3 Global 500m SIN Grid V006. NASA EOSDIS Land Processes DAAC. Accessed 2021-02-23 from <https://doi.org/10.5067/MODIS/MCD12Q1.006>. Accessed February 23, 2021.
4. Wan, Z., Hook, S., Hulley, G. (2015). MOD11A2 MODIS/Terra Land Surface Temperature/Emissivity 8-Day L3 Global 1km SIN Grid V006. NASA EOSDIS Land Processes DAAC. Accessed 2021-02-23 from <https://doi.org/10.5067/MODIS/MOD11A2.006>. Accessed February 23, 2021
5. Didan, K. (2015). MOD13A2 MODIS/Terra Vegetation Indices 16-Day L3 Global 1km SIN Grid V006. NASA EOSDIS Land Processes DAAC. Accessed 2021-02-23 from <https://doi.org/10.5067/MODIS/MOD13A2.006>. Accessed February 23, 2021.
6. Fick, S.E. and R.J. Hijmans, 2017. WorldClim 2: new 1km spatial resolution climate surfaces for global land areas. International Journal of Climatology 37 (12): 4302-4315.
7. de Sousa, L. M., Poggio, L., Batjes, N. H., Heuvelink, G. B. M., Kempen, B., Riberio, E., and Rossiter, D.: SoilGrids 2.0: producing quality-assessed soil information for the globe, SOIL, 7, 217-240, https://doi.org/10.5194/soil-7-217-2021.
