# Supplementary material for: Geostatistical analysis of active human cysticercosis: Results of a large-scale study in 60 villages in Burkina Faso
Source: PLoS Negl Trop Dis. 2023 Jul 26;17(7):e0011437. doi: 10.1371/journal.pntd.0011437 (PMC10370738; doi:10.1371/journal.pntd.0011437)
Supplement: S2 Table — (DOCX) [file pntd.0011437.s007.docx]

**S2 Table. Summary measures for environmental data extracted for** individual**- and village-level datasets**

|  | Dataset | | |
| --- | --- | --- | --- |
| Parameter | Individual-level ($n$ = 3550) |  | Village-level ($n$ = 60) |
| Elevation (m) |  |  |  |
| Mean (SD) | 296 (20.1) |  | 296 (19.8) |
| Median [Min, Max] | 291 [258, 352] |  | 291 [264, 346] |
| Evaporation (mm/month) |  |  |  |
| Mean (SD) | 184 (5.47) |  | 184 (5.48) |
| Median [Min, Max] | 185 [169, 192] |  | 185 [170, 191] |
| Land temperature, day (°C) |  |  |  |
| Mean (SD) | 32.2 (2.58) |  | 32.2 (2.56) |
| Median [Min, Max] | 31.9 [27.0, 40.4] |  | 31.9 [28.3, 39.7] |
| Land temperature, night (°C) |  |  |  |
| Mean (SD) | 24.2 (0.52) |  | 24.2 (0.47) |
| Median [Min, Max] | 24.2 [22.4, 27.7] |  | 24.2 [22.6, 25.6] |
| NDVI |  |  |  |
| Mean (SD) | 0.37 (0.068) |  | 0.37 (0.066) |
| Median [Min, Max] | 0.36 [0.25, 0.61] |  | 0.36 [0.28, 0.61] |
| Precipitation (mm/month) |  |  |  |
| Mean (SD) | 168 (2.66) |  | 168 (2.66) |
| Median [Min, Max] | 168 [159, 174] |  | 168 [160, 173] |
| Soil pH (%) |  |  |  |
| Mean (SD) | 6.32 (0.19) |  | 6.32 (0.14) |
| Median [Min, Max] | 6.30 [2.70, 6.70] |  | 6.31 [6.06, 6.69] |
| Soil silt (%) |  |  |  |
| Mean (SD) | 26.7 (3.39) |  | 26.7 (3.18) |
| Median [Min, Max] | 26.4 [10.0, 41.1] |  | 26.4 [18.8, 37.6] |
| Soil sand |  |  |  |
| Mean (SD) | 52.0 (5.65) |  | 52.0 (5.39) |
| Median [Min, Max] | 52.1 [22.2, 69.6] |  | 52.0 [37.2, 67.3] |
| Soil clay (%) |  |  |  |
| Mean (SD) | 21.1 (3.94) |  | 21.2 (3.77) |
| Median [Min, Max] | 22.0 [9.7, 30.8] |  | 22.3 [13.9, 28.2] |
| Distance to the nearest river (km) |  |  |  |
| Mean (SD) | 3.86 (2.95) |  | 3.85 (2.86) |
| Median [Min, Max] | 3.00 [0, 15.0] |  | 3.6 [0.60, 14.5] |

SD: standard deviation
