# Supplementary material for: Geostatistical analysis of active human cysticercosis: Results of a large-scale study in 60 villages in Burkina Faso
Source: PLoS Negl Trop Dis. 2023 Jul 26;17(7):e0011437. doi: 10.1371/journal.pntd.0011437 (PMC10370738; doi:10.1371/journal.pntd.0011437)
Supplement: S3 Table — (DOCX) [file pntd.0011437.s008.docx]

**S3 Table. Fitted generalized linear models for the individual-level and village-level datasets**

| Model | Model-level | Parameter | Estimate | 95%CI | $p$-value |
| --- | --- | --- | --- | --- | --- |
| $\mathcal{M}_{S1}$ | Individual | Intercept | -9.54 | [-27.1;7.19] | 0.275 |
|  |  | Precipitation (mm/month) | 0.11 | [0.028;-0.19] | 0.008 |
|  |  | Distance to the nearest river (km) | -0.088 | [-0.16;-0.019] | 0.015 |
|  |  | Land temperature, night (°C) | -0.47 | [-0.84;-0.090] | 0.015 |
| $\mathcal{M}_{S2}$ | Village | Intercept | -23.9 | [-37.6;-10.7] | <0.001 |
|  |  | Precipitation (mm/month) | 0.12 | [0.045;0.21] | 0.002 |
|  |  | Distance to the nearest river (km) | -0.085 | [-0.16;-0.013] | 0.026 |
| $\mathcal{M}_{S1}$ | Individual | Intercept | -23.9 | [-37.4;-10.9] | <0.001 |
|  |  | Precipitation (mm/month) | 0.12 | [0.046;0.20] | 0.002 |
|  |  | Distance to the nearest river (km) | -0.080 | [-0.15;-0.011] | 0.027 |

95%CI: 95% confidence interval
