# Supplementary figures and images for: Geostatistical analysis of active human cysticercosis: Results of a large-scale study in 60 villages in Burkina Faso
Source: PLoS Negl Trop Dis. 2023 Jul 26;17(7):e0011437. doi: 10.1371/journal.pntd.0011437 (PMC10370738; doi:10.1371/journal.pntd.0011437)

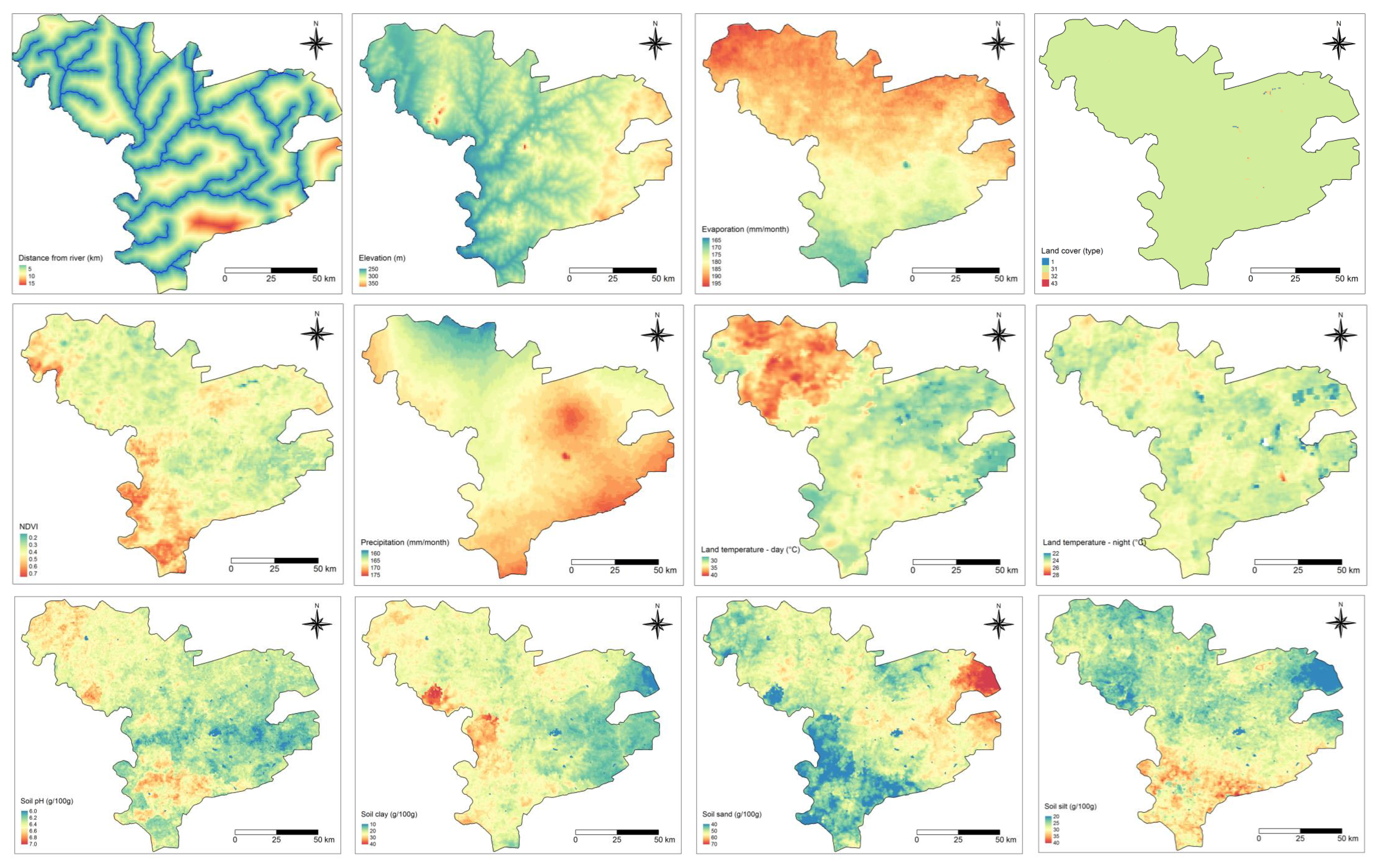


**S1 Fig. Maps of the environmental variables at the study area**

Supplement: S1 Fig — (DOCX) [file pntd.0011437.s010.docx]

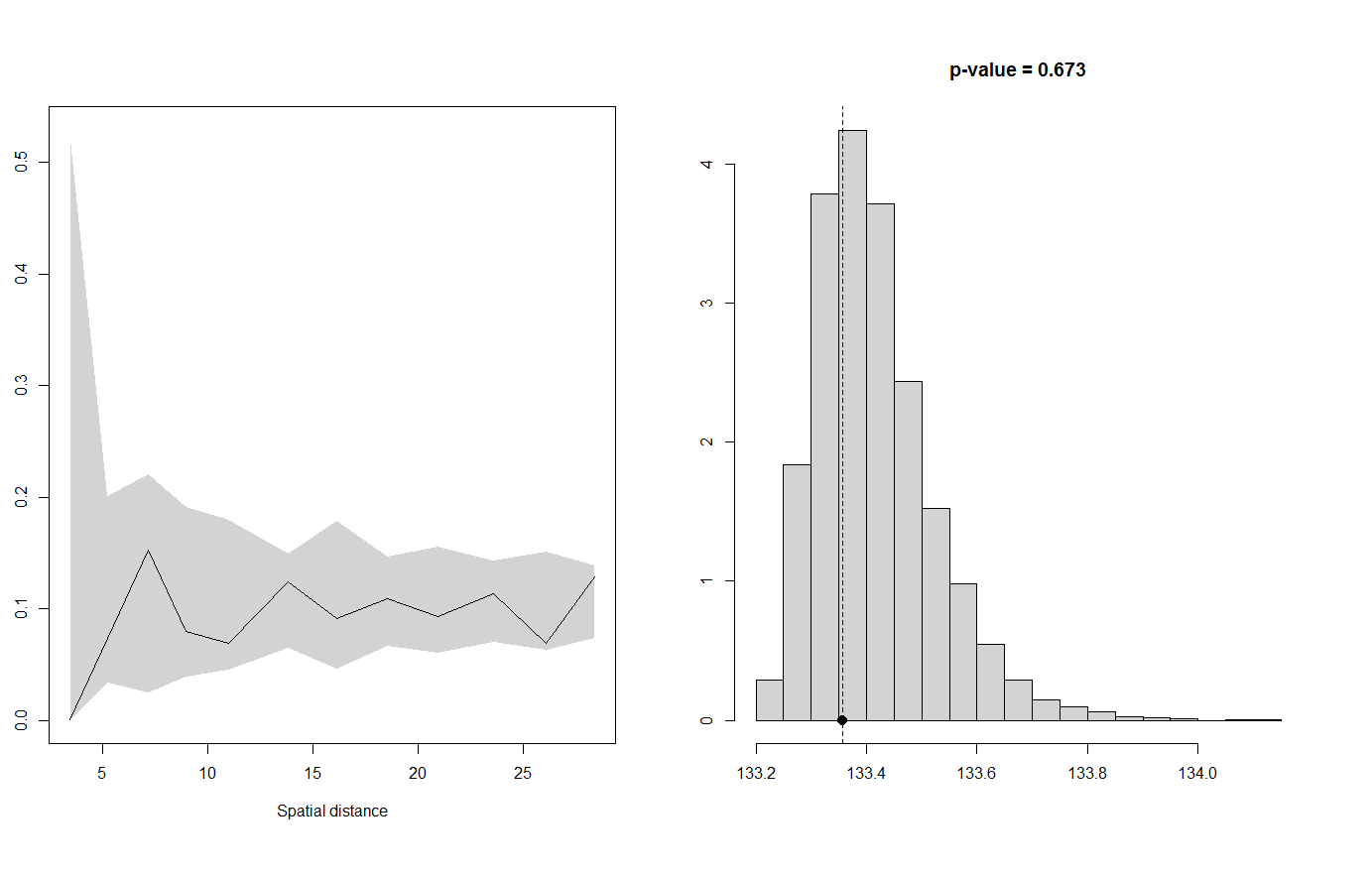


**S6 Fig. Testing for residual spatial correlation for the village-level data (distance in km**

Supplement: S6 Fig — (DOCX) [file pntd.0011437.s015.docx]

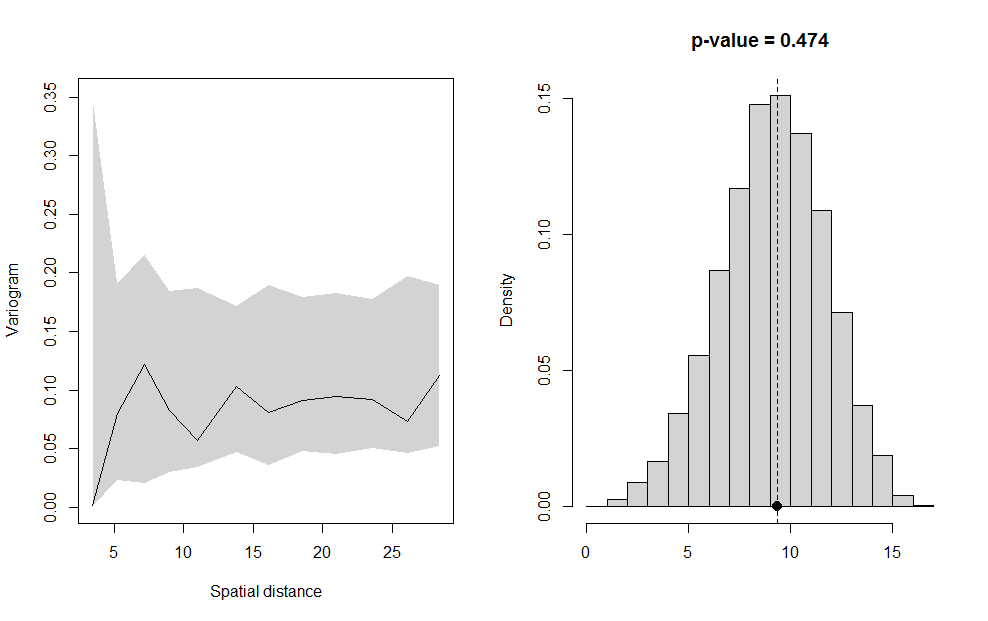


**S7 Fig. Test the fit of the final geostatistical model for the village-level data (distance in km)**

Supplement: S7 Fig — (DOCX) [file pntd.0011437.s016.docx]
