# Supplementary material for: Geostatistical analysis of active human cysticercosis: Results of a large-scale study in 60 villages in Burkina Faso
Source: PLoS Negl Trop Dis. 2023 Jul 26;17(7):e0011437. doi: 10.1371/journal.pntd.0011437 (PMC10370738; doi:10.1371/journal.pntd.0011437)
Supplement: S2 Fig — Relationship between village-level environmental variables and empirical logit outcome (Part A). (Green = linear regression, blue = general additive model with penalized smoother). (DOCX) [file pntd.0011437.s011.docx]

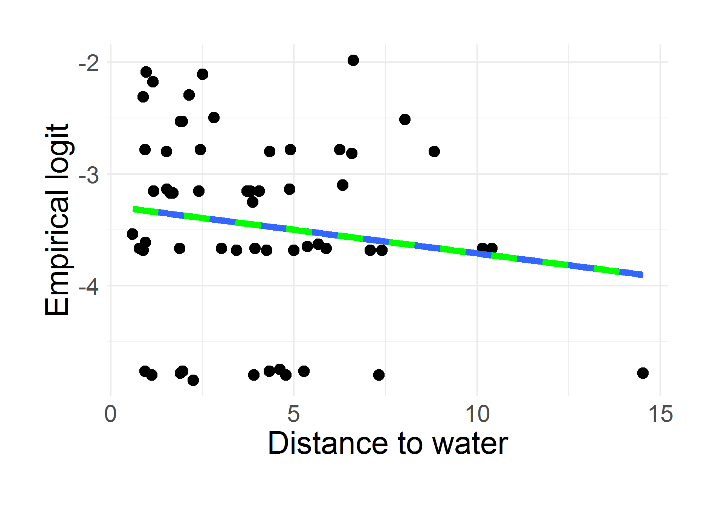

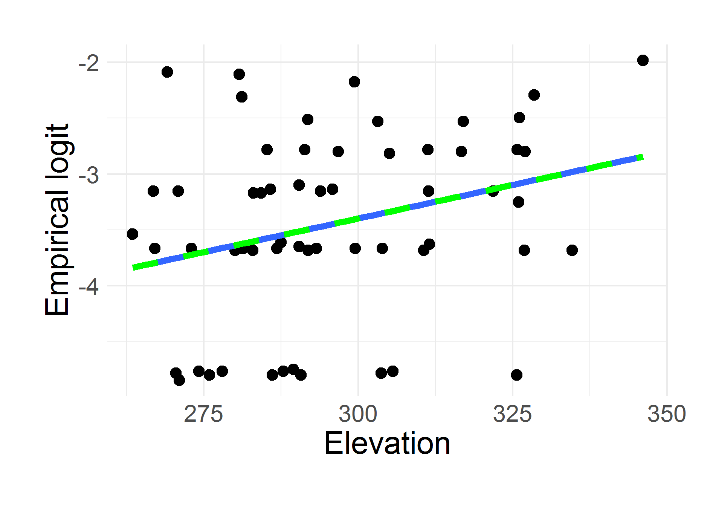

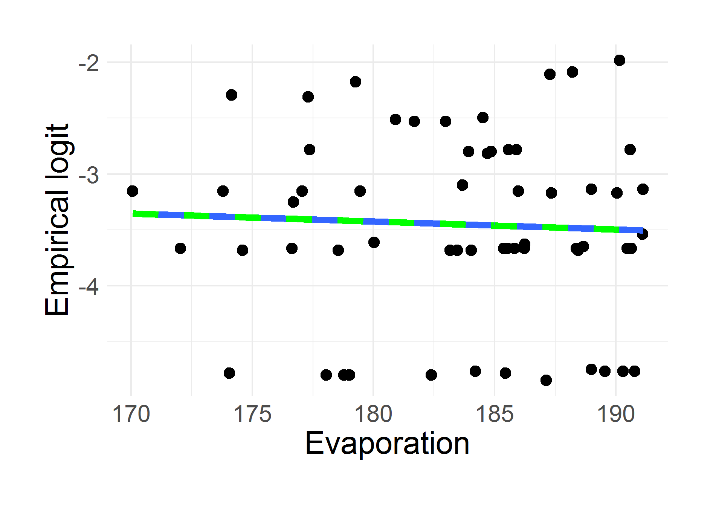

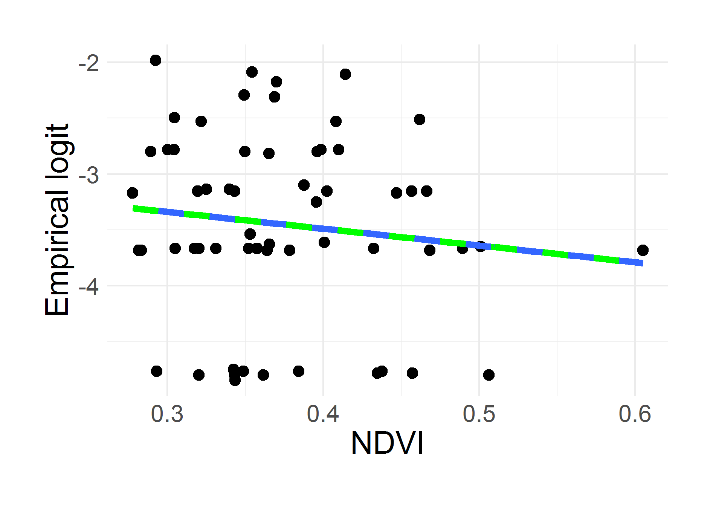

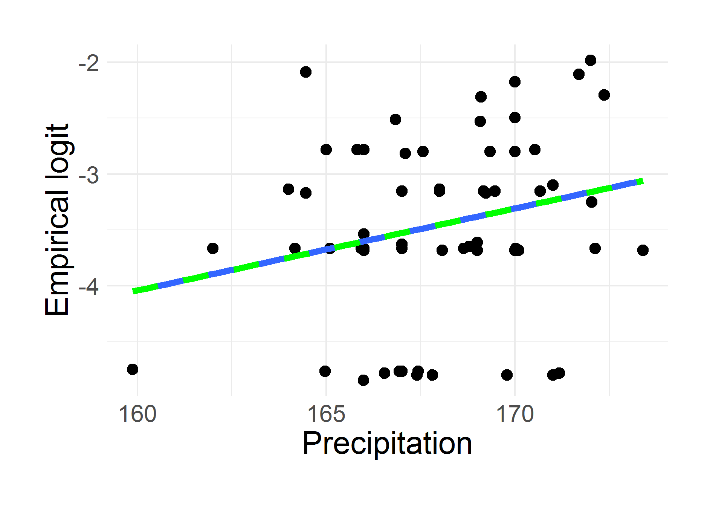

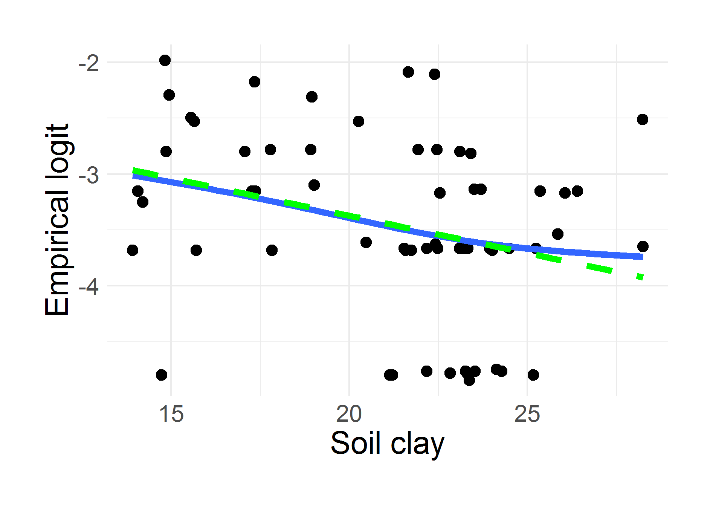
 **S2 Fig. Relationship between village-level environmental variables and empirical logit outcome (Part A).**(Green = linear regression, blue = general additive model with penalized smoother)
